# Reconstituting development of pancreatic intraepithelial neoplasia from primary human pancreas duct cells

**DOI:** 10.1038/ncomms14686

**Published:** 2017-03-08

**Authors:** Jonghyeob Lee, Emily R. Snyder, Yinghua Liu, Xueying Gu, Jing Wang, Brittany M. Flowers, Yoo Jung Kim, Sangbin Park, Gregory L. Szot, Ralph H. Hruban, Teri A. Longacre, Seung K. Kim

**Affiliations:** 1 Department of Developmental Biology, Stanford University School of Medicine, 279 Campus Drive, Beckman Center B300, Stanford, California 94305, USA; 2 UCSF Transplantation Surgery, University of California, San Francisco, San Francisco, California 94143, USA; 3 The Sol Goldman Pancreatic Cancer Research Center, Department of Pathology, Johns Hopkins University School of Medicine, Baltimore, Maryland 21287, USA; 4 Department of Pathology, Stanford University School of Medicine, Stanford, California 94305, USA

## Abstract

Development of systems that reconstitute hallmark features of human pancreatic intraepithelial neoplasia (PanINs), the precursor to pancreatic ductal adenocarcinoma, could generate new strategies for early diagnosis and intervention. However, human cell-based PanIN models with defined mutations are unavailable. Here, we report that genetic modification of primary human pancreatic cells leads to development of lesions resembling native human PanINs. Primary human pancreas duct cells harbouring oncogenic *KRAS* and induced mutations in *CDKN2A*, *SMAD4* and *TP53* expand *in vitro* as epithelial spheres. After pancreatic transplantation, mutant clones form lesions histologically similar to native PanINs, including prominent stromal responses. Gene expression profiling reveals molecular similarities of mutant clones with native PanINs, and identifies potential PanIN biomarker candidates including Neuromedin U, a circulating peptide hormone. Prospective reconstitution of human PanIN development from primary cells provides experimental opportunities to investigate pancreas cancer development, progression and early-stage detection.

## Abstract (web summary)

Models of human pancreatic intraepithelial neoplasia (PanIN) development do not exist. Here, the authors induce oncogenic *KRAS* and mutations in *CDKN2A*, *SMAD4* and *TP53* in primary human pancreatic cells to generate a PanIN model that recapitulates molecular and pathologic features of native PanINs.

Pancreatic ductal adenocarcinoma (PDA) typically presents at late stages with dismal overall survival. By contrast, fortuitous detection of early-stage disease localized to the pancreas can lead to curative treatment. Based on retrospective analysis of human tissue samples, the investigators postulate that a series of genomic mutations accumulate in pancreatic exocrine cells leading to dysplastic lesions called pancreatic intraepithelial neoplasia, PanIN1 and PanIN2, then PanIN3 (carcinoma *in situ*) before progressing to invasive PDA^1^,^2^. Among mutated genes, *KRAS* has been most closely associated with PDA and its precursors, with over 90% of PanINs and PDAs harbouring oncogenic *KRAS* mutations^3^. Loss-of-function mutations at high prevalence in ‘tumour suppressors' encoded by *CDKN2A* (90–95%), *SMAD4* (49–55%) and *TP53* (50–84%) are coupled to protein loss and also tightly linked to PDA formation^4^,^5^. In human PDA, mutations in only one or two of these genes is infrequent; more commonly, three or four mutations are found in combination^4^. This suggests that multiple genomic alterations are required to initiate PDA development or progression. Collectively, mutations in *KRAS*, *CDKN2A*, *SMAD4* and *TP53* have been dubbed ‘driver mutations' for human PDA formation^4^,^6^.

Findings from genetically engineered mouse models (GEMM) support this genetic PDA progression model. These findings include the observation that expression of oncogenic *Kras* alleles is sufficient to induce development of PanIN-like lesions in GEMM^7^ and, depending on the developmental stage of *Kras* induction, to induce invasive PDA. The frequency and severity of invasive phenotypes can be increased in these genetic mouse models when oncogenic *Kras* expression is combined with other driver mutations or with experimental pancreatitis^8^. Despite impressive advances in genetically engineered mouse models of PDA development, there is no evidence that healthy human pancreatic cells can form PanIN or invasive PDA when similar driver mutations are introduced. Given the translational value of human PDA modelling, several groups attempted to generate human PanIN or PDA models using various cell sources such as an immortalized human ductal cell line^9^, human embryonic stem cells^10^ or induced pluripotent stem cells^11^, or organoids derived from PDA patients^12^. However, none of these prior examples systematically introduced driver mutations in human pancreatic exocrine cells from healthy donors and reconstituted the features of human PanIN or PDA.

Here we report that recapitulating driver mutations in primary human pancreatic ductal cells reconstitutes development of lesions resembling PanINs. Lentiviral gene delivery combined with CRISPR-Cas9 genome-editing systems achieves permanent alterations in *KRAS*, *CDKN2A*, *SMAD4* and *TP53* in primary human duct cells. Cloned immortalized cells grow as epithelial monolayer spheres in three-dimensional culture. On orthotopic transplantation into adult mouse pancreas, these cells form structures with multiple cellular and molecular features of PanINs that do not progress after 6 months to invasive PDA. Thus, we generated a unique system to develop stable human PanIN-like lesions prospectively from healthy human pancreatic ductal cells. This will provide a robust experimental system for investigation of developmental, genetic and signalling mechanisms underlying formation of PanINs from healthy human duct cells.

## Results

### Genetic modification of purified primary human duct cells

**Figure 1:**
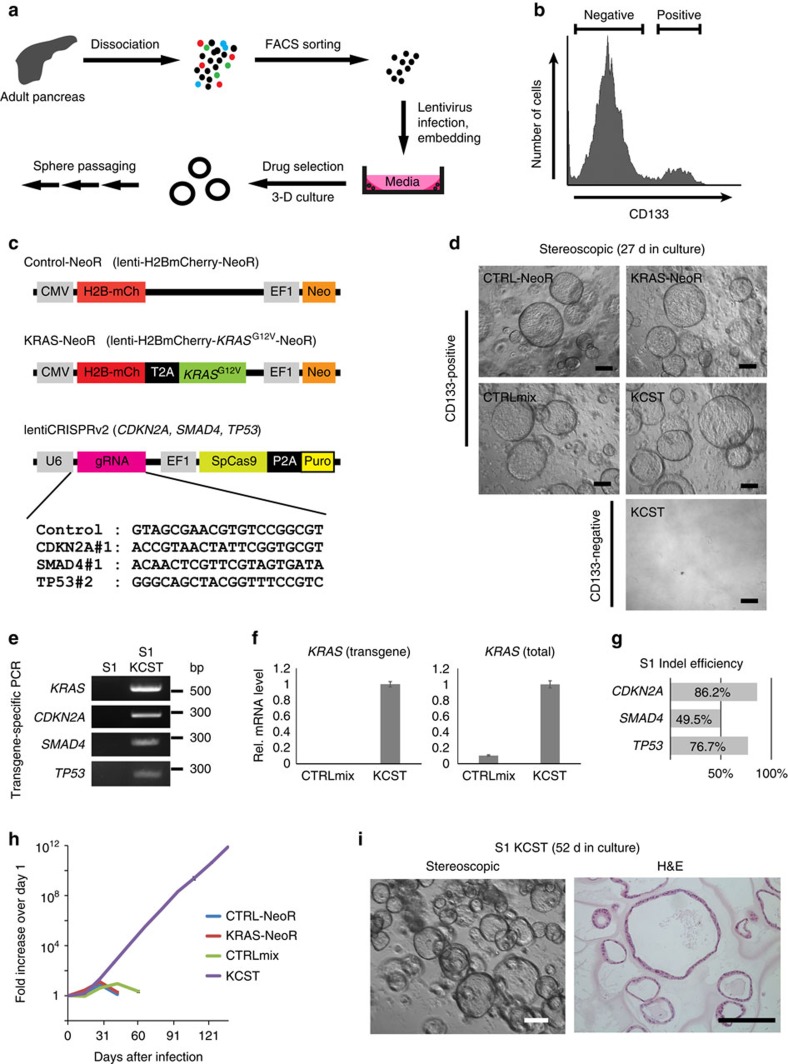
Oncogenic *KRAS* expression and tumour suppressor inactivation immortalizes purified primary human duct cells. (**a**) Schematic diagram summarizing experimental procedures. (**b**) FACS histogram of the dissociated human adult pancreas stained with antibody specific for CD133. Results are representative of three independent experiments. (**c**) Schematics of lentiviral constructs used and sgRNA sequences for the construction of lentiCRISPRv2. (**d**) Representative images of spheres cultured for 27 days from CD133^+^ ductal cells infected with combinations of lentiviruses (CTRL-*NeoR*, Control-*NeoR* alone; KRAS-*NeoR*, KRAS-*NeoR* alone; CTRLmix, Control-*NeoR* and lentiCRISPRv2-Control; KCST, KRAS-*NeoR* plus lentiCRISPRv2 against CDKN2A#1, SMAD4#1 and TP53#2). (**e**) Genomic DNA PCR for confirming the presence of lentiviral transgenes in uninfected (S1; Supplementary Table 1) and infected (S1 KCST) spheres. bp=base pair. (**f**) Relative mRNA expression level of oncogenic *KRAS* transgene (left) and the transgene plus endogenous *KRAS* (right); *n*=2. (**g**) Indel efficiency of each indicated genomic locus assessed by TIDE analysis, (**h**) Quantification of the total cell number in each cell passage of CD133^+^ cells infected with indicated combinations of lentiviruses. Data are presented as fold increase over day 1. (**i**) Representative stereoscopic and haematoxylin and eosin (H&E) staining images of S1 KCST spheres after 52 days in culture. Error bars=s.d., scale bars, 200 μm.

To investigate whether the genetic and cellular hallmarks of human PanIN development can be reconstituted in purified normal human pancreas cells, we used FACS to isolate pancreatic exocrine cells from human cadaveric donors without known pancreatic diseases (Fig. 1a and Supplementary Table 1)^13^. FACS fractionation using CD133 antibody separated CD133^+^ cells expressing ductal markers like KRT19 and CAR2, and CD133^−^ cells that include acinar and endocrine cells (Fig. 1b)^13^. We were unable to expand CD133^−^ cells *in vitro* (Fig. 1d, ‘CD133^−^'), precluding studies of PanIN or PDA development from human acinar cells. By contrast, duct cells survived and expanded as monolayer epithelial spheres cultured in Matrigel in a defined medium without serum or feeder cells up to 40 days, and then ceased growth (Fig. 1h)^13^. We found that purified CD133^+^ cells could be infected with lentivirus harbouring the gene encoding the fluorescent protein H2B-mCherry, and genes conferring resistance to the drugs puromycin or neomcyin (Supplementary Fig. 1A and Methods section), indicating that genetic modification of primary duct cells using lentiviral methods is feasible.

Oncogenic *Kras* activation alone in pancreatic epithelial cells is sufficient to initiate PDA development in mice. However, *Kras* activation alone infrequently leads to development of invasive PDA, while combination with mutations in *Cdkn2a*, *Smad4* or *Trp53* can enhance the speed or frequency of progression to invasive disease in GEMM^8^. Moreover, it is not known whether such genetic changes are also sufficient to induce PDA development in human pancreatic exocrine cells. To study this question, we constructed lentiviruses expressing oncogenic *KRAS*^*G12V*^ and lentiCRISPRv2 viruses (see Methods section) encoding the Cas9 nuclease and single guide RNA (sgRNAs) against the genomic loci for *CDKN2A*, *SMAD4* and *TP53* (KCST viruses, where K=*KRAS*, C=*CDKN2A*, S=*SMAD4* and T=*TP53*; Fig. 1c). Purified CD133^+^ cells were infected with (1) control lentivirus producing H2B-mCherry and a neomycin resistance gene (Control-*NeoR*; CTRL), (2) virus expressing *KRAS*^*G12V*^ and *NeoR* (*KRAS-NeoR*; KRAS), (3) a mixture of control viruses (Control-*NeoR* and lentiCRISPRv2-Control; CTRLmix) or (4) a combination of KCST viruses, and then grown in sphere cultures (Fig. 1d). Concurrently, CD133^−^ cells were also infected with KCST viruses. All the infection combinations ((1)–(4) above) of CD133^+^ cells grew as spheres in 2 weeks and continued to grow up to 4 weeks after passaging; by contrast, CD133^−^ cells, including acinar cells^13^, failed to grow and expand (Fig. 1d,h). Genomic DNA PCR and quantitative real-time PCR (qRT–PCR) with the KCST virus-infected spheres confirmed the presence of all four transgenes (Fig. 1e) and mRNA expression of the oncogenic *KRAS*^*G12V*^ (Fig. 1f). PCR amplification and DNA sequencing of the target genomic regions, followed by Tracking of Indels by DEcomposition (TIDE) analysis^14^, revealed high-efficiency genome editing with our lentiCRISPR reagents in *CDKN2A*, *SMAD4* and *TP53* loci, evidenced by a high prevalence of insertion or deletion (indel) mutations (49.5–86.2%; Fig. 1g and Supplementary Fig. 1D). Thus, our approach induced genetic and targeted genomic modifications of four PDA-associated ‘driver' genes. We observed exponential growth of the KCST primary spheres over 5 months, while the spheres transduced with lentiviruses encoding Control-*NeoR*, *KRAS-NeoR*-Neo alone or a mixture of control viruses (CTRLmix) failed to expand beyond 30 days after infection (Fig. 1h). Haematoxylin–eosin staining of growing KCST spheres showed epithelial monolayers composed of cuboidal cells (Fig. 1i and Supplementary Fig. 1C). The cytoplasm of these cells failed to stain with Alcian blue, which detects acid mucin production, a characteristic feature of PanINs (Supplementary Fig. 1C). These data indicate that our lentiviral reagents efficiently induce genetic and genomic modifications of the PDA-associated genes (KCST) in purified normal human primary pancreatic ductal cells, and induce their immortalization.

### Development of PanIN-like lesions after transplantation

**Figure 2:**
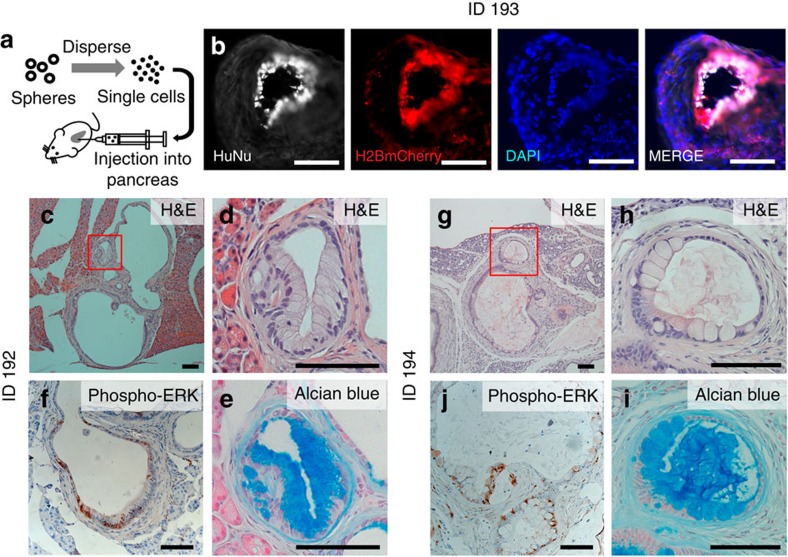
Genetically modified human ductal cells develop PanIN-like lesions after orthotopic transplantation. (**a**) Schematic diagram of the orthotopic transplantation procedure. (**b**) immunohistochemical analyses of a PanIN-like structure in transplanted animal ID 193 with the human nuclei-specific antibody (HuNu, white) and H2B-mCherry fluorescence (red) along with 4,6-diamidino-2-phenylindole (DAPI) nuclear staining (blue). (**c**) Haematoxylin and eosin (H&E) staining of the PanIN-like structures found in transplanted animal ID 192 with magnified view of red-boxed area in **d**. (**e**) Alcian blue staining of tissue section adjacent to that shown in **d**. (**f**) Immunohistochemical analysis with phospho-ERK antibody. (**g**) H&E staining of the transplanted mouse ID 194 with magnified view of the red-boxed area in **h**. (**i**) Alcian blue staining of tissue section adjacent to that shown in **h**. (**j**) Immunohistochemical analysis with phospho-ERK antibody. Scale bars, 100 μm.

To assess the tumorigenic potential of the transduced primary human ductal spheres, we enzymatically dispersed and orthotopically transplanted the KCST spheres into the pancreas splenic lobe of immunocompromised NOD *scid IL2rγ*^*null*^ (NSG) mice (Fig. 2a and Methods section). For up to 6 months after transplantation in all mice transplanted (*n*=3), we found extraductal PanIN-like structures in the splenic lobe (Fig. 2 and Supplementary Fig. 1B). Histological analyses of the entire splenic lobe of each host pancreas revealed that all three injections had produced lesions surrounded by a prominent desmoplastic reaction (Fig. 2 and Supplementary Table 2). Many lesions comprised tall columnar-shaped cells with basally located nuclei and mucinous cytoplasm, the typical features^1^ of human PanIN1 (Fig. 2c,d,g,h). Alcian blue staining confirmed the presence of acid mucins (Fig. 2e,i). Activated oncogenic *KRAS* is associated with increased phospho-extracellular signal-regulated kinase (ERK), and immunohistochemistry detected increased phospho-ERK in these lesions at levels and in patterns comparable to those in native human PanINs (Fig. 2f,j and Supplementary Fig. 3)^15^. By contrast, little to no phospho-AKT was detected in PanIN-like lesions (Supplementary Fig. 3), consistent with prior reports that native human PanIN lesions show weak or non-detectable phospho-AKT immunoreactivity^16^. mCherry fluorescence further confirmed that the human-induced PanIN-like (hiPanIN hereafter) structures were derived from transduced human spheres (indicating lentiviral H2B-mCherry transgene coexpression with *KRAS*^*G12V*^; Fig. 1c); immunostaining with antibodies detecting human nuclear antigen (HuNu) and human mitochondria, antigens expressed exclusively in human cells, confirmed the human donor origin of hiPanIN cells (Fig. 2b and Supplementary Figs 4D and 5). By contrast, host mouse cells comprised the adjacent fibroblastic desmoplasia. Collectively, these data indicate that alterations of *KRAS*, and targeting *CDKN2A*, *SMAD4* and *TP53* (KCST), are sufficient to transform normal human ductal cells into human PanIN1. We have not observed evidence of invasive or metastatic lesions in any of the transplanted mice with hiPanIN cells (see below).

### Development of PanIN2-like lesions with *ERBB2* in hiPanINs

**Figure 3:**
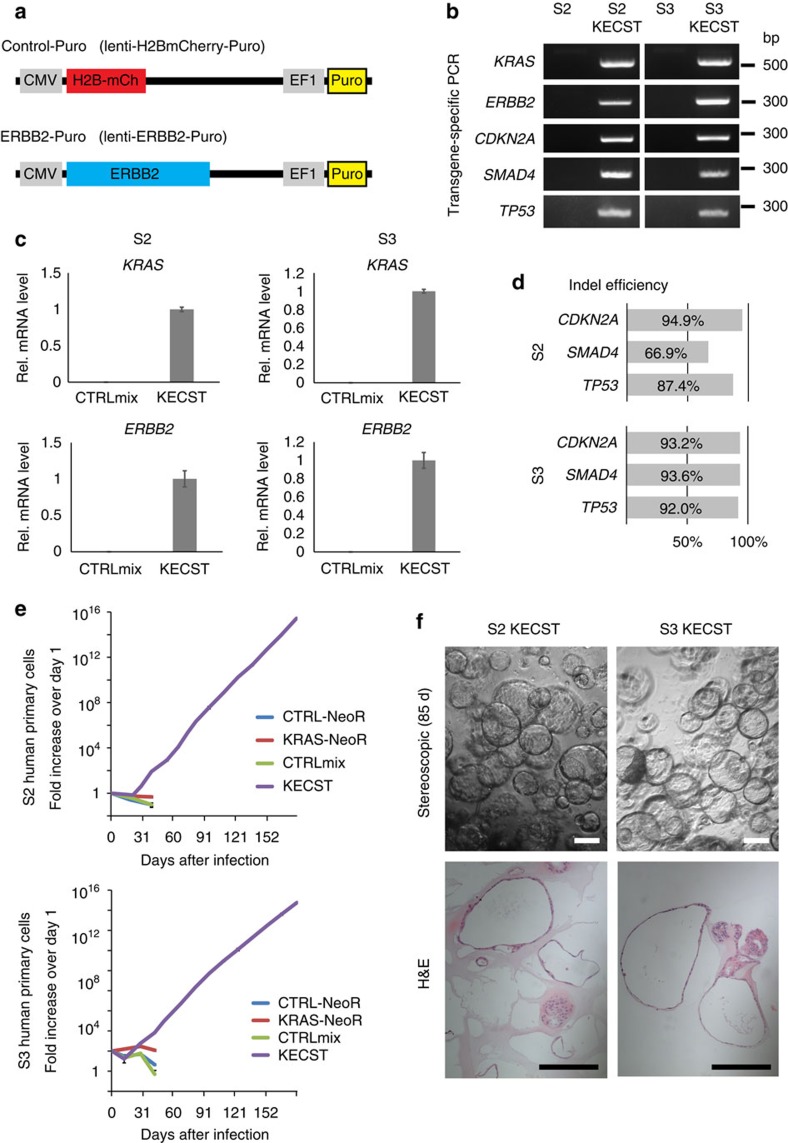
Expression of *ERBB2* and oncogenic *KRAS* along with tumour suppressor inactivation immortalizes purified primary human duct cells. (**a**) Schematic of lentiviral constructs encoding H2B-mCherry and human *ERBB2*. (**b**) Genomic DNA PCR confirming the presence of lentiviral transgenes in uninfected (S2 and S3) and infected (S2 KECST and S3 KECST) spheres. (**c**) Relative mRNA expression level of oncogenic *KRAS* and *ERBB2* transgenes in S2 KECST (left) and S3 KECST (right) spheres; *n*=2. Error bars=s.d. (**d**) Indel efficiency of each indicated genomic locus assessed by TIDE analysis. (**e**) Quantification of the total cell number in each cell passage of CD133^+^ cells infected with indicated combinations of lentiviruses. (**f**) Representative stereoscopic and H&E staining images of KECST spheres after 85 days in culture. Scale bars, 200 μm.

**Figure 4:**
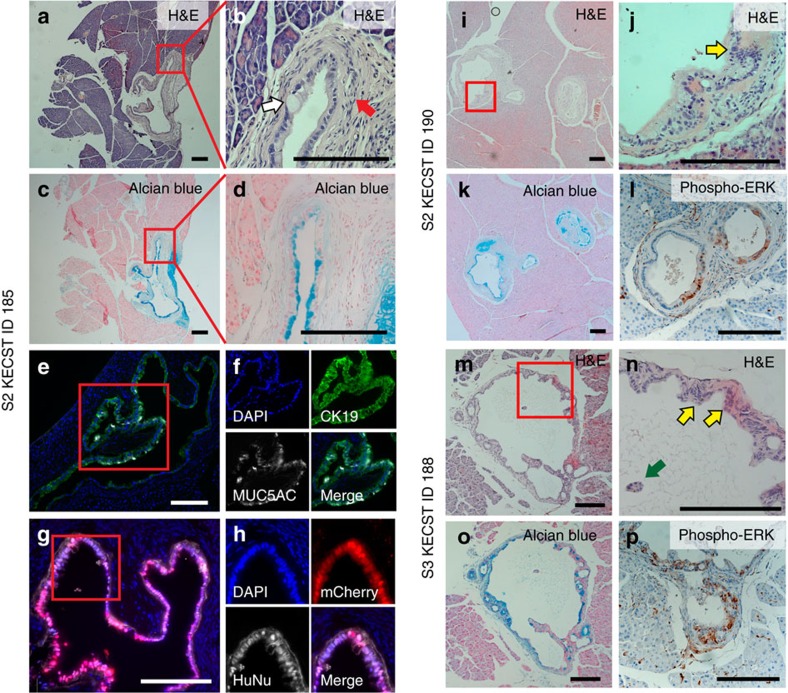
Development of PanIN2-like lesions after orthotopic transplantation of transduced primary human ductal KECST spheres. (**a**) Haematoxylin and eosin (H&E) staining of transplanted mouse pancreas ID 185 with S2 KECST spheres. (**b**) Magnified image of the red-boxed area in **a**. White arrow indicates mucinous cytoplasm and red arrow points to the encasing stromal cells. (**c**) Alcian blue staining of the adjacent section of **a** and magnified view of the red-boxed area in **d**. (**e**) Immunohistochemistry of the PanIN-like structure in ID 185 with antibody detecting CK19 (green) or antibody detecting MUC5AC (white) along with 4,6-diamidino-2-phenylindole (DAPI) nuclear staining (blue). Magnified images in different fluorescent channels of the red-boxed area are shown in **f**. (**g**) Immunohistochemistry with human nuclear-specific antibody (HuNu, white) and mCherry fluorescence (red) and magnified view of the red-boxed area in **h**. (**i**) H&E staining of mouse ID 190 transplanted with S2 KECST spheres. Magnified view of the red-boxed area is shown in **j**. Alcian blue staining and phospho-ERK immunohistochemistry result are shown in **k**,**l**, respectively. (**m**) H&E staining of mouse ID 188 transplanted with S3 KECST spheres. Magnified view of red-boxed area is shown in **n**. Alcian blue staining and phospho-ERK immunohistochemistry result are shown in **o**,**p**, respectively. Yellow and green arrows indicate representative abnormal nuclei and an epithelial cell cluster found in the lumen, features of human PanIN2. Scale bars, 200 μm.

Although not considered a ‘driver' of PDA development, the oncogene *ERBB2* is frequently overexpressed in human PanIN and invasive PDA^5^. To assess whether overexpression of *ERBB2* along with KCST mutations can promote development of clearly invasive PDA from primary human ductal cells, we sorted CD133^+^ cells from two independent donors (S2 and S3; Supplementary Table 1) and infected with lentiviruses encoding *ERBB2* (Fig. 3a) as well as oncogenic *KRAS*^*G12V*^ and lentiCRISPRs targeting *CDKN2A*, *SMAD4* and *TP53* loci (five alterations abbreviated ‘KECST'). Genomic DNA PCR and qRT–PCR confirmed the presence of all transgenes and expression of mRNAs encoding *KRAS*^*G12V*^ and *ERBB2*, respectively (Fig. 3b,c). TIDE analysis revealed high-efficiency genome editing using our lentiCRISPR reagents (66.9–93.9%; Fig. 3d and Supplementary Fig. 2A and B). Similar to KCST-transduced spheres, KECST-transduced spheres also grew exponentially over 6 months (Fig. 3e), indicating immortalization of the transduced KECST spheres. H&E staining revealed that monolayer cuboidal cells comprise growing KECST spheres, similar to features found in KCST spheres (Fig. 3f). After orthotopic transplantation of KECST spheres (*n*=9; Supplementary Table 2), histological analyses revealed that eight of nine transplanted pancreata had PanIN-like structures (Fig. 4, Supplementary Figs 2C and 4A–C and Supplementary Table 2). However, we did not observe evidence of invasive or metastatic tumours. Alcian blue staining and immunohistochemical analysis for MUC5AC further confirmed the presence of acid mucins in KECST-derived hiPanINs (Fig. 4c–f,k,o). Immunostaining also revealed increased phospho-ERK signals in these lesions, consistent with activation of KRAS (Fig. 4l,p and Supplementary Fig. 3). In addition, we found that these lesions maintained characteristic features of ducts, including production of cytokeratin 19 (CK19) detected by immunolabelling (Fig. 4e,f). We also confirmed that these lesions were derived from transduced human spheres by positive mCherry fluorescence and immunodetection of human nuclear antigen and human mitochondria (Fig. 4g and Supplementary Figs 4D, 5 and 6). In a subset of KECST hiPanINs, we observed clear features of PanIN2-like architecture and cytology, including papillary structures, nuclear crowding, enlarged hyperchromatic nuclei, nuclear pleomorphism and pseudostratification (Fig. 4i,j,m,n). Collectively, these data indicate that *ERBB2* misexpression in KECST hiPanIN cells leads to development of PanIN2 features, but is not sufficient to produce invasive PDA.

### Cloned hiPanIN cells produce PanIN-like lesions

**Figure 5:**
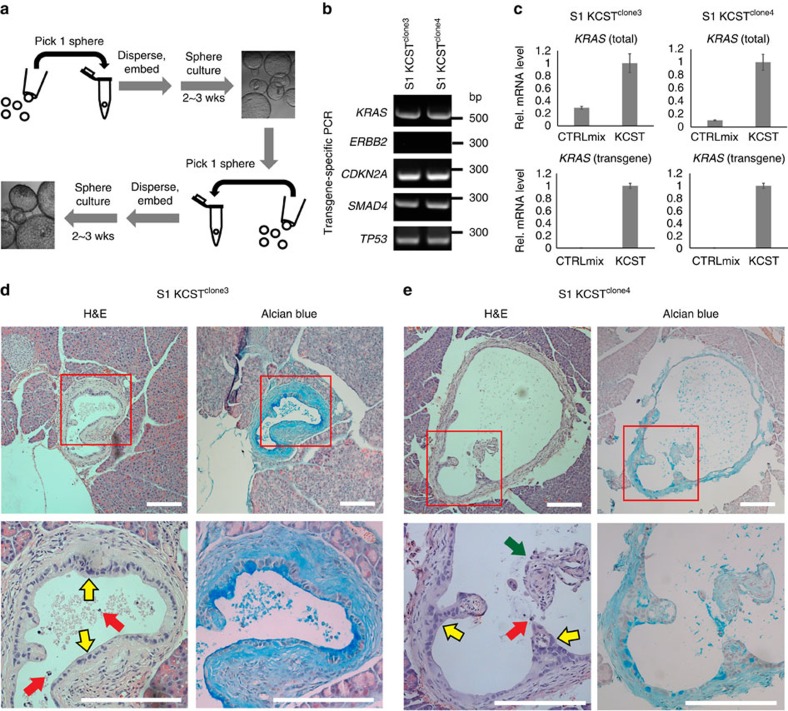
Clones with defined genomic mutations form PanIN-like lesions but not PDA. (**a**) Schematic of the sphere clone isolation procedure. See Methods for details. (**b**) Genomic DNA PCR confirming the presence of lentiviral transgenes in clone 3 (left) and clone 4 (right). (**c**) Relative mRNA expression level of oncogenic *KRAS* transgene (bottom) and the transgene plus endogenous *KRAS* (top) of clone 3 (left) and clone 4 (right). Error bars=s.d.; *n*=2. (**d**,**e**) H&E (left) and Alcian blue (right) staining of the transplanted pancreas ID 199 with clone 3 in **d** and ID 207 with clone 4 in **e**. Yellow arrows indicate representative abnormal nuclei, red and green arrows indicate necrotic cells and epithelial cell clusters found in the lumen, features of human PanIN2 and 3. Scale bars, 200 μm.

Prior studies suggest that PanINs and PDA can develop as clonal lesions intermixed with normal epithelium surrounded by a stromal compartment with acellular matrix and non-epithelial cells including fibroblasts, immune cells and endothelial cells^17^,^18^,^19^. While the efficiency of gene targeting in KCST or KECST spheres was high, the primary spheres we transplanted orthotopically represented mixtures of normal and genetically altered duct cells, not clones (Figs 2c,g and 4i). Thus, we isolated clones and repeated our transplantation studies to investigate if development of cloned hiPanIN cells modifies histopathological outcomes (Fig. 5a). We isolated 21 clones from growing KCST- and KECST-transduced spheres derived from three independent donors (S1–S3; Supplementary Table 1 and Fig. 5a). Consistent with the mixed nature of transduced spheres, genomic DNA PCR, TIDE analyses and genomic DNA sequencing revealed unique genomic alterations in each clone (Supplementary Figs 4E, 7A,B, 8A–D and 10A,B and Supplementary Table 3). Among these, we isolated four clones for further analysis. One clone expressed *KRAS*^*G12V*^ and had homozygous frameshift mutations in *CDKN2A* and *TP53* loci (S2 KCT^clone3^) (Supplementary Fig. 7A,B and Supplementary Table 3). Three clones had *KRAS*^*G12V*^ expression and homozygous frameshift mutations in *CDKN2A*, *SMAD4* and *TP53* loci (S1 KCST^clone3^, S1 KCST^clone4^ and S2 KCST^clone8^) (Fig. 5b,c, Supplementary Figs 8 and 10, and Supplementary Table 3). In addition, we generated a clone with *ERBB2* by infecting S1 KCST^clone4^ with *ERBB2*-encoding lentivirus (Fig. 3a) to generate S1 KECST^clone4^ (Supplementary Fig. 9C). Distinct single or double peaks in TIDE analyses confirmed that each hiPanIN cell line was clonal (Supplementary Figs 7A, 8A,B and 10A). Lists of candidate off-target sites for individual sgRNAs used were generated using an off-target site prediction tool (see Methods section). TIDE analyses in each clone demonstrated that off-target effects did not correspond with phenotypes observed in hiPanIN cell clones (Supplementary Table 4 and Supplementary Figs 11–14).

After orthotopic transplantation, we observed development of PanIN-like lesions in cloned hiPanIN cell lines with KCST and KECST genotypes—S1 KCST^clone3^, S1 KCST^clone4^, S1 KECST^clone4^ and S2 KCST^clone8^ (Table 1, Fig. 5d,e and Supplementary Figs 9 and 10). For each of these clones, orthotopic transplantation produced lesions with features characteristic of ‘late'-stage PanIN2 and PanIN3 lesions, including prominent nuclear abnormalities, mitotic figures, cribriforming, budding of small clusters of epithelial cells into the lumen or luminal necrosis (Fig. 5d,e and Supplementary Fig. 9A,B)^1^. By contrast, we observed development of normal duct-like structures in mice transplanted with S2 KCT^clone3^ cells (Table 1 and Supplementary Fig. 7C), suggesting an essential role for *SMAD4* loss in hiPanIN development. Up to 4 months after transplantation, we did not observe evidence of invasive or metastatic PDA in any case. Thus, our studies reveal the potential of cloned hiPanIN cell lines to develop into stable lesions with characteristic features of early-stage PDA, including PanIN2 and PanIN3. In addition, we demonstrate that oncogenic *KRAS*^*G12V*^ expression together with null mutations in *CDKN2A*, *SMAD4* and *TP53* in previously healthy human ductal cells is sufficient to produce lesions resembling human PanINs, but not invasive PDA within the framework of our experiments.

### Genetic modification of HPDE cells induces PDA development

**Figure 6:**
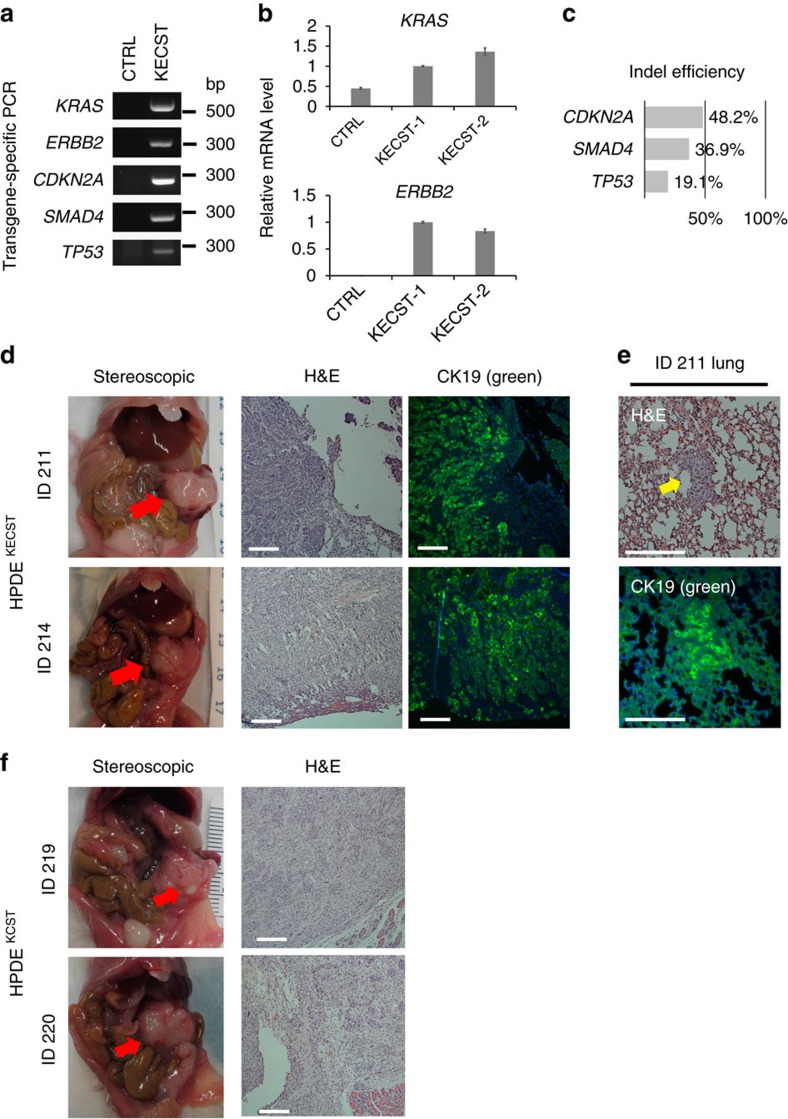
Genetic modification of human ductal cell line HPDE induces invasive PDA development. (**a**) Genomic DNA PCR for assessing the presence of lentiviral transgenes in HPDE cells. (**b**) Relative mRNA expression level of oncogenic *KRAS* and *ERBB2* transgene. Error bars=s.d.; *n*=2. (**c**) Indel efficiency of each indicated genomic locus assessed by TIDE analysis. (**d**) Stereoscopic and representative haematoxylin and eosin (H&E) and anti-CK19 (green) immunostaining images of the tumours formed in the transplanted pancreas with HPDE^KECST^. Red arrows indicate tumour nodules. (**e**) Representative H&E staining of lung with metastatic cells found in ID 211. The metastatic cells are CK19^+^ (green, bottom). (**f**) Stereoscopic and representative H&E staining images of the tumours formed in the transplanted pancreas with HPDE^KCST^. Scale bars, 200 μm.

Are the genetic modifications used here sufficient to convert ductal cells into invasive PDA? To address this, in parallel studies we engineered KCST or KECST modifications in human pancreatic duct epithelial (HPDE) cells, a duct cell line derived using human papilloma virus-16 to produce E6/E7, viral oncoproteins that destabilize p53 protein^20^. While the parental HPDE cell line does not form tumours after orthotopic transplantation, expression of oncogenic *KRAS*^*G12V*^ and *ERBB2* combined with short hairpin RNA-mediated knockdown of *CDKN2A* and *SMAD4* produced lesions resembling invasive PDA after transplantation^9^. We used the same lentiviral and lentiCRISPR reagents used for generating hiPanIN cells (Figs 1c and 3a) to generate HPDE cells harbouring genomic mutations in *CDKN2A*, *SMAD4* and *TP53* and expressing *KRAS*^*G12V*^ and *ERBB2* (HPDE^KECST^), confirmed by genomic DNA PCR and qRT–PCR (Fig. 6a,b). TIDE analysis revealed relatively lower genome-editing efficiency than in primary pancreas duct cells (19.1–48.2%; Fig. 6c and Supplementary Fig. 15A). After transplantation in NSG mice, dispersed HPDE control cells failed to grow (*n*=2). By contrast, all mice grafted with HPDE^KECST^ cells developed multiple heterogeneous nodules in the pancreas within 8 weeks (Supplementary Table 5 and Fig. 6d). Histological analysis of the nodules revealed complex, moderate to poorly differentiated or poorly differentiated adenocarcinoma (Fig. 6d). Immunohistochemical analysis confirmed that tumours maintained expression of the ductal marker CK19 (Fig. 6d, CK19, green). Moreover, lung nodules were CK19^+^, consistent with a metastatic HPDE^KECST^ origin (Fig. 6e). Similarly, we found the tumour development 8 weeks after orthotopic transplantation of HPDE cells harbouring genomic mutations in *CDKN2A*, *SMAD4* and *TP53* and expressing *KRAS*^*G12V*^ (HPDE^KCST^; Fig. 6f and Supplementary Table 5), indicating that overexpression of *ERBB2* is dispensable for invasive tumour development in this model. To ensure that invasive tumour formation did not reflect off-target effects by lentiCRISPR reagents, we designed additional sets of sgRNAs to target *CDKN2A*, *SMAD4* and *TP53* (CDKN2A#3, SMAD4#2 and TP53#3) to generate HPDE^KECST2^ and HPDE^KCST2^ (Supplementary Fig. 15B–E). When orthotopically transplanted into NSG mice, both HPDE^KECST2^ and HPDE^KCST2^ cells formed invasive adenocarcinoma in 8 weeks (Supplementary Table 5 and Supplementary Fig. 15F–H). Collectively, these data indicate that our lentiviral reagents successfully induce genetic and genomic alterations of the PDA-associated genes, and that such driver mutations are sufficient to generate HPDE cell-derived lesions resembling invasive or metastatic PDA after orthotopic transplantation.

### Molecular comparison of hiPanINs to native PanIN and PDA

**Figure 7:**
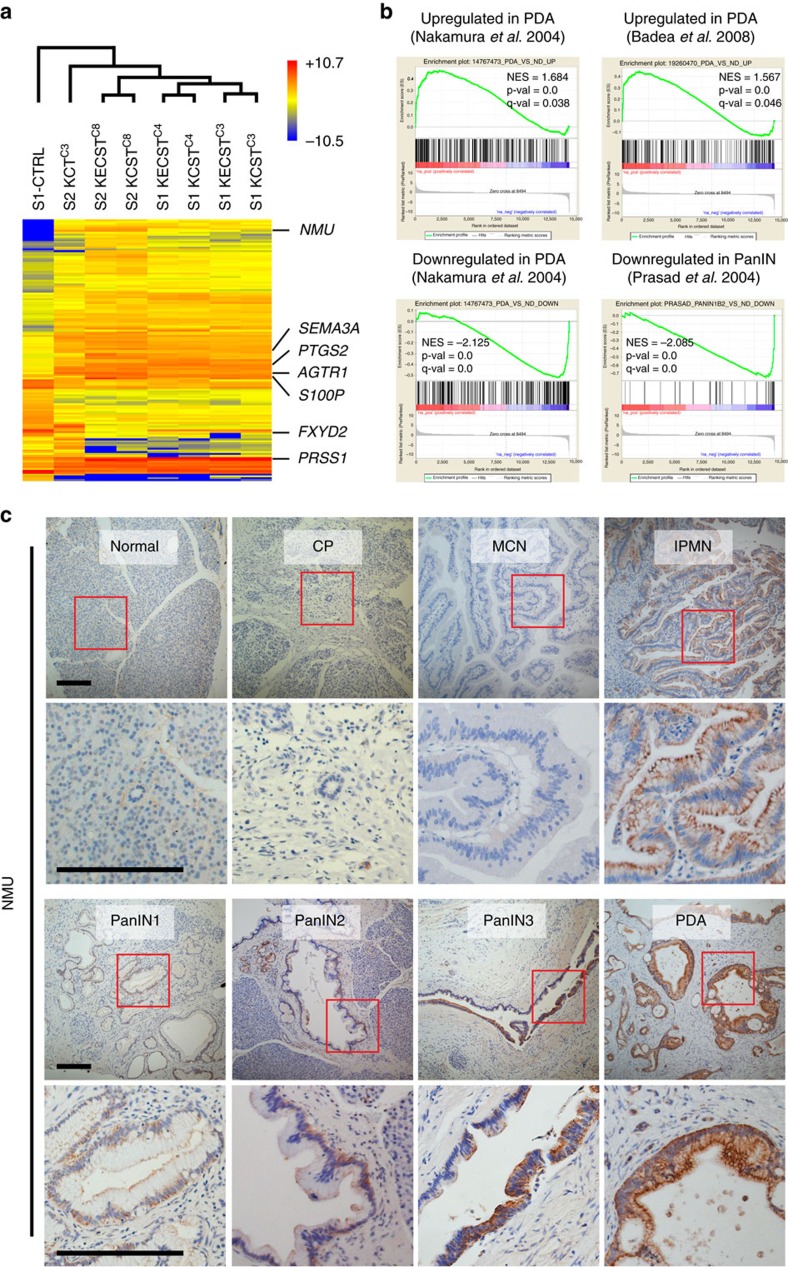
Global gene expression profiling of hiPanIN clones reveals their molecular similarities with native PanIN and PDA. (**a**) RNAseq data presented as a hierarchically clustered heatmap. (**b**) Gene set enrichment analysis on RNAseq data with genesets compiled with publically available PDA and PanIN microarray data. NES, normalized enrichment score; p-val, nominal *P* value; q-val, false discovery rate *q*-value. (**c**) NMU immunohistochemical analyses on tissues with various pancreatic lesions. Results are representative of three to six independent experiments. Scale bars, 200 μm.

To define the transcriptome of hiPanIN clones, we performed RNAseq analysis using KCT, KCST and KECST clones along with donor-matched control ductal sphere cells (S1 CTRL, S1 KCST^clone3^, S1 KECST^clone3^, S1 KCST^clone4^, S1 KECST^clone4^, S2 KCT^clone3^, S2 KCST^clone8^ and S2 KECST^clone8^; Fig. 7a). We discovered that 92 genes were upregulated and 48 downregulated more than fourfold in KCST and KECST clones compared with control cells grown in spheres (Supplementary Data 1). Of the 92 upregulated, six genes (*AGTR1*, *EBF4*, *MXRA5*, *PRSS1*, *PTGS2* and *S100P*) were previously reported to be induced in human PanINs by microarray analyses or immunohistochemistry^21^,^22^,^23^,^24^,^25^. Also, 23 upregulated genes including *FN1*, *SEMA3A* and *GATA3* were shown previously, using mass spectrometry, RNA expression profiling and western blotting, to be induced in PDA (Supplementary Data 1). Among 48 downregulated genes, *FXYD2* was previously reported to have reduced expression in two independent PanIN microarray analyses^21^,^22^, while five genes had been previously reported to have reduced expression in human PDA (Supplementary Data 1). Gene set enrichment analysis (GSEA) of our RNAseq data revealed statistically significant induction of genes involved in epithelial–mesenchymal transition, G-to-M checkpoint and apoptosis (Supplementary Table 6), hallmark signatures related to cancer development. To further assess molecular similarities between our cultured hiPanIN clones and clinical PanIN or PDA specimen, we generated custom human PanIN/PDA gene sets with publically available microarray data and performed GSEA on our RNAseq data. As expected, we observed statistically significant enrichment of our RNAseq data in three published PanIN and PDA genesets (Fig. 7b). Collectively, these data suggest that our cultured hiPanIN clones show molecular similarities to clinical PanIN and PDA specimens.

Neuromedin U (NMU) was originally identified as a neuropeptide that induces uterine smooth muscle contraction^26^; recently, NMU has been suggested to be a circulating hormone that suppresses pancreatic islet insulin secretion^27^. Misexpression of pancreatic NMU protein has been previously reported in advanced human PDA, but not in precursor lesions like PanINs^28^. Our RNAseq analyses revealed NMU as one of the most highly elevated genes in all hiPanIN clones (Fig. 7a and Supplementary Data 1), suggesting that NMU misexpression may initiate in PDA precursor stages. To address this possibility, we performed NMU immunohistochemistry on appropriate clinical tissue sections (Fig. 7c; see Methods section). We did not detect NMU immunoreactivity in cases of normal pancreas (four out of four), chronic pancreatitis (three of three) or mucinous cystic neoplasms (MCN, five of five). However, we detected NMU protein production in intraductal papillary mucinous neoplasms (IPMNs; four of six cases), PanINs (six of six cases) and PDA (six of six cases; Fig. 7c). In each case of PanINs and IPMN, 50% of the pancreatic lesions labelled with NMU-specific antibody. In PanINs we observed this percentage of immunostaining in all PanIN grades 1–3. In contrast, more than 95% of individual PDA lesions were NMU-positive, suggesting that NMU immunoreactivity and expression increases as the precursor lesions progress to PDA. Thus, our hiPanIN models identify a potential marker of precancerous PanIN and IPMN lesions including NMU.

## Discussion

To advance genetic and developmental studies of human PDA initiation, we have built systems to reconstitute PanIN development by purifying and genetically modifying primary human pancreatic duct cells. Studies of the earliest experimentally accessible stages of human PDA development are relevant for generating diagnostic tools to detect PDA when it remains curable by surgical resection, and here we focused on producing human systems that recapitulate hallmarks of human PanIN development and cancer genetics. This human-centred approach also reflects the growing appreciation of differences between human and mouse pancreas development and pancreas cancer biology^17^,^29^. Here, we show that four genetic modifications in normal human pancreatic duct cells are sufficient to generate cells that can reconstitute stable PanIN-like structures without progression to invasive PDA in an orthotopic transplant model. The reproducibility of this system is further enhanced by our ability to clone multiple hiPanIN cell lines from independent human donors. These findings address several basic questions about human PDA development, and based on our ability to induce, clone and cryopreserve cell lines that generate PanIN-like lesions, provide a robust experimental system for investigating the developmental biology and genetics of human PDA. RNAseq analysis further supported the molecular similarity of cultured hiPanIN clones with human PanIN/PDA, and revealed potential PanIN markers including NMU. Although further studies are needed to demonstrate the clinical relevance of the biomarker candidates identified here, including NMU, our findings demonstrate the potential usefulness of systematic genetic targeting in our hiPanIN systems for identifying human PDA biomarkers to advance detection of early-stage disease.

Recent studies have reported efforts to reconstitute PDA development in cultured human cells^11^,^12^, but the resources and conclusions from these studies are distinct from those reported here in several important ways. Kim *et al*.^11^ reported reprogramming of cells derived from PDA resections to generate induced pluripotent stem cell lines, and teratomas formed by these after transplantation harboured early PanIN-like lesions that later progressed to invasive carcinoma. Since their starting material derived from resected advanced PDA, the genetic changes necessary for PanIN development in this system were not identified. Boj *et al*.^12^ established human organoid cultures from unfractionated human pancreatic clusters and resected PDA samples from subjects with late-stage disease. However, systematic alterations of human PDA driver genes have not been performed in normal organoids; thus, the genetics of PanIN and PDA development were not directly addressed in that study. Huang *et al*.^10^ generated human pancreatic exocrine spheroids from human ES cells and introduced oncogenic *KRAS*^*G12V*^ and *p53*^*R175H*^ mutations to model PDA progression. Lesions produced in that study, however, did not recapitulate typical molecular and histological features of human PanIN or PDA either in culture or in transplantation settings. By contrast, we purified human pancreatic ductal cells from disease-free donors, and introduced driver mutations to address their necessity for PanIN or PDA formation in normal pancreatic duct cells. This strategy generated cloned hiPanIN cell lines with defined genetic changes that generate stable PanIN-like structures derived from duct cells.

It remains unclear why hiPanIN cells develop into PanIN-like lesions but not to PDA. In mice, oncogenic *KRAS* expression in exocrine cells is sufficient to promote both mouse PanIN (mPanIN) and PDA, when induced during embryonic pancreas development. However, when oncogenic *KRAS* expression is induced in adult mouse pancreas to model the adult onset of nearly all human PDA development, mPanIN or PDA failed to develop, even with additional deletion of *p16*^*INK4A*^ or *Trp53* allele^30^,^31^. When chronic pancreatitis was induced, such mice showed development of mPanIN or PDA^30^,^31^, suggesting that both genetic and non-genetic acquired conditions may be needed to model invasive PDA development with hiPanIN cells. Our attempts to induce caerulein-mediated chronic pancreatitis in mice transplanted with hiPanIN clones have not produced invasive PDA, likely due to defective immune systems in NSG mice^32^. Consistent with this, survey of innate immune cells including macrophages (MAC-2 and F4/80), neutrophil (Ly6B.2) and mast cells (Toluidine Blue O) revealed the absence of these cells around the hiPanIN lesions, and weak or no immunoreactivity with phospho-signal transducer and activator of transcription 3 (STAT3) antibody was observed in the lesions (J.Lee and S.Kim, unpublished observations). Thus, it remains unclear whether chronic pancreatitis, inflammatory cells (which are mostly absent in NSG mice used for orthotopic transplants here) or other factors thought to promote PanIN-to-PDA progression could influence development of invasive-stage PDA-like lesions by hiPanIN cells. Studies on the pace of genetic and cellular changes in human pancreatic cancer suggest that the time from mutations that initiate PanIN formation to development of invasive disease and metastasis may span decades^18^,^33^. Therefore, compared to mice, the latency of PDA development by human cells may be intrinsically longer. The cell of origin may also be attributable to the resistance of hiPanIN to become PDA. Studies in mice have led to the hypothesis that both acinar cells and ductal cells have the potential to generate invasive PDA but via different precancerous developmental routes^34^. Therefore, it is possible that ductal cells in pancreas are more resistant to become PDA than acinar cells. Unfortunately, human pancreatic acinar cells did not grow in culture: thus, the PanIN/PDA-forming potential of human pancreatic acinar cells in our system remains unclear. hiPanIN progression to PDA may also require additional mutations or mutation combinations. In contrast to the spheres derived from primary human cells, the KCST combination of genetic changes promoted development of metastatic tumours by HPDE cells, a human pancreatic ductal line immortalized by human papilloma virus-16 E6/E7 that normally does not form tumours in xenotransplant models^9^,^20^. The parental HPDE line has multiple chromosomal deletions, in addition to expression of the E6/E7 oncogenes^35^; thus, future studies should test the hypothesis that additional genetic modification of hiPanIN^KCST^ and hiPanIN^KECST^ duct cells could promote development of invasive or metastatic phenotypes. Alternatively, the tempo and order of mutations during native PanIN development may be important for PanIN-to-PDA progression of our hiPanIN models. As our hiPanIN model permits experimental modulation of the tempo and order of gene mutation, future studies should test this possibility. In summary, although the lack of progression to PDA limits our understanding of human pancreatic carcinogenesis, the stability of PanIN-like lesions in our system provides a unique platform and experimental advantages for identifying factors, mutations and conditions that promote invasive PDA development from late-stage PanINs.

Findings from our study also suggest that important intercellular or signalling interactions in PDA development are recapitulated by hiPanIN cells after orthotopic transplantation. A characteristic feature of PanIN and PDA development is the prominent fibrotic response found encasing hyperplastic or dysplastic pancreatic epithelial cells, and we observed this in all our orthotopic transplants. We also observed striking changes of hiPanIN cell nuclear and cytoplasmic morphology induced by transplantation, supporting the possibility that relevant reciprocal signalling interactions occur between hiPanIN cells and surrounding stroma. Although the contribution of stroma in PDA development, growth and survival appears well established^36^, it remains unclear whether cues from stroma or other non-epithelial components are also required for normal-to-native human PanIN development. Our hiPanIN cells are generated in defined medium without serum or feeder cells; thus, modulation of these cells with purified signalling factors or non-epithelial human cells should be feasible, and could accelerate studies of human stromal–epithelial signalling in PanIN and PDA progression. In our transplantation studies with the parental S1–3 hiPanIN cells, which represent a mixed population of mutant and normal cells, we did not observe production of lesions with features of ‘more advanced' PanIN3, which is thought to precede development of invasive PDA. One interpretation of this finding is that cells with less than four mutations form low-grade PanINs and are found more frequently than those with all four in clonal populations. Consistent with this possibility, we found that transplantation of specific *cloned* cell lines (S1 clones) produced lesions with features of PanIN2 and PanIN3. Together, these findings support the hypothesis that genetic heterogeneity of epithelial cells within individual PDA lesions could affect histopathological development^19^. In summary, targeting a small number of genes in primary pancreatic ductal cells from human donors without known pancreas disease was sufficient to induce clones capable of generating PanIN-like structures. This unique resource should be useful for identifying human PDA biomarkers to advance detection of early-stage disease, and for investigating fundamental genetic or signalling mechanisms that promote PanIN development into invasive PDA.

## Methods

### Primary human ductal cell preparation

Human islet-depleted cell fractions were obtained from healthy organ donors deceased due to acute traumatic or anoxic death from UCSF Diabetes Center (University of California, San Francisco, CA). Donor samples used for this study are listed in Supplementary Table 1. On receipt, the human islet-depleted cell fractions were washed with PBS, trypsinized with 0.05% Trypsin-EDTA solution (Life Technologies) for 5 min and quenched with five volumes of FACS buffer (10 mM EGTA, 2% fetal bovine serum in PBS). Cells were collected by centrifugation and further digested in 1 U ml^−1^ dispase solution (Life Technologies) containing 0.1 mg ml^−1^ DNaseI in PBS on a nutating mixer at 37 °C for 30 min. PBS washing was performed after each enzymatic digestion step. After centrifugation, the cell pellet was resuspended in FACS buffer and passed through a 40-μm-cell strainer (BD Biosciences, Bedford, MA). Dispersed cells were stained with biotin-conjugated CD133 antibodies (clone AC133 and 293C3; Miltenyi Biotec, Auburn, CA), followed by allophycocyanin-conjugated streptavidin (eBioscience, San Diego, CA) for sorting pancreatic ductal cells using a FACSAria II (BD Biosciences, Bedford, MA). A total of 8 × 10^5^, 4 × 10^6^ and 1 × 10^6^ of CD133^+^ cells were collected from S1, S2 and S3 donor samples, respectively, and 2–5 × 10^5^ cells were used for each lentiviral infection combination (Control-*NeoR*, *KRAS*-*NeoR*, CTRLmix, KCST or KECST).

### HPDE cell culture

HPDE cell line (H6c7) was obtained as a gift from Dr Ming-Sound Tsao (University Health Network, Ontario, Canada)^20^,^35^. In short, cells were maintained and grown in KGM Bullet Kit (Lonza Group Ltd, , Basel, Switzerland; Clonetics KBM, cat. no. CC-3111) by replacing media twice a week. When passaging, cells were trypsinized with 0.5% Trypsin-EDTA solution (Invitrogen, Carlsbad, CA) and neutralized with 0.1% trypsin inhibitor (Invitrogen).

### Construction and preparation of lentiviral vectors

To construct KRAS-Neo lentivirus, H2B-mCherry insert was PCR amplified from pDual-H2B-mCherry shuttle vectors^37^ using primers containing *Xba*I and T2A plus *Xho*I sites, respectively (5′-GAAGATTCTAGAGCCACCATGCCTGAACCCTCTAAGTCTG-3′ and 5′-AGTCATCTCGAGGGGGCCGGGGTTCTCCTCCACGTCGCCGCAGGTCAGCAGGCTGCCGCGGCCCTCGCCGCTGCCGCTGCGCTTGGCGCGCTTGTACAGCTCGTCCATGCCGCC-3′), and the PCR product was digested with *Xba*I and *Xho*I. Human *KRAS*^*G12V*^ was PCR amplified from pBABE puro K-Ras V12 (a gift from William Hahn; Addgene plasmid no. 9052) using primers containing *Xho*I and *Not*I restriction enzyme sites, respectively (5′-AACCCCGGCCCCCTCGAGATGACTGAATATAAACTTGTGGTA-3′ and 5′-ATCCTTGCGGCCGCTTACATAATTACACACTTTGTCTT-3′), and the PCR product was digested with *Xho*I and *Not*I. pCDH-CMV-MCS-EF1-Neo (System Biosciences, Mountain View, CA) was linearized with *Xba*I and *Not*I, and ligated with two inserts. Control-Neo and Control-Puro were constructed similarly by ligating PCR-amplified H2Bm-Cherry insert into pCDH-CMV-MCS-EF1-Neo and pCDH-CMV-MCS-EF1-Puro (System Biosciences), respectively. To construct ERBB2-Puro, human ERBB2 insert was PCR amplified from HER2/PLNCX2 vector, a gift from Dr Paul Chiao^9^, using two primers (5′-CCATAGAAGATTCTAGAGCTAGCGCTAGCGCTACCGGACT-3′ and 5′-tcgcagatccttcgcggccgcACCTACAGGTGGGGTCTTTC-3′). pCDH-CMV-MCS-EF1-Puro was linearized with *Not*I and *Nhe*I, and ligated with Gibson Assembly Cloning Kit (New England Biolabs, Ipswich, MA). LentiCRISPR reagents were constructed using lentiCRISPRv2 (a gift from Feng Zhang, Addgene plasmid no. 52961), according to the recommended protocol published in Addgene.org. All constructs were sequence verified and the primer sequence information is shown in Supplementary Table 7. Lentiviruses were produced by transfecting HEK 293T cells with the vector, pMD2.G and psPAX2 (a gift from Didier Trono, Addgene plasmid no. 12259 and 12260) DNAs in 5:2:3 ratio with TurboFect (Thermo Fisher Scientific, Waltham, MA). Lentiviral particles were precipitated and concentrated with PEG-it (System Biosciences) by centrifugation at 1,500*g* for 30 min at 4 °C.

### Lentiviral infection and post-infection culture

To infect HPDE cells, media were replaced with fresh media supplemented with 8 μg ml^−1^ polybrene (Sigma-Aldrich, St Louis, MO) and lentiviruses were added directly into the media. Media were replaced after 3 days and puromycin selection began at a concentration of 0.5 μg ml^−1^ for 5 days.

Sorted human primary ductal cells were washed twice with Advanced DMEM/F-12 media (Invitrogen) and resuspended 2–5 × 10^5^ cells in 300 μl of sphere culture media—Advanced DMEM/F-12 media supplemented with recombinant human epidermal growth factor (50 ng ml^−1^; Sigma), rhR-spondin I (500  ng ml^−1^; R&D Systems, Minneapolis, MN), recombinant human fibroblast growth factor 10 (50 ng ml^−1^; R&D Systems), recombinant mouse Noggin (100 ng ml^−1^; R&D Systems), 10 mM nicotinamide in PBS and Pen/Strep. The resuspended cells were placed in a well of 24-well Ultra Low Cluster Plate (Costar 3473, Corning, New York) and mixed with lentivirus for suspension infection overnight at 37 °C. Next day, infected cells were washed twice with PBS and resuspended with 120 μl sphere culture media. Two hundred microlitres of growth factor-reduced Matrigel (BD Biosciences) was then added and the mixture was placed around the bottom rim of each well of 12-well plate. After solidification at 37 °C for 60 min, each well was overlaid with 2 ml of sphere culture media. Media were replaced twice a week and the spheres were collected after 2 to 3 weeks for passaging. Static images of spheres were collected using Zeiss Axiovert 200 inverted microscope (Carl Zeiss, Germany). For collecting spheres, 1 ml of 3U ml^−1^ dispase (Life Technologies) solution containing 0.1 mg ml^−1^ DNaseI in PBS was added in each well and the Matrigel was mechanically disrupted by pipetting and incubated at 37 °C for 1 h. The released spheres were collected, washed twice with PBS and used for subsequent applications. For passaging spheres, the collected spheres were trypsinized at 37 °C for 5 min followed by quenching with fetal bovine serum. The dispersed cells were then used for cell counting with a haemocytometer or were plated for sphere culture as described above.

To isolate sphere clones, individual spheres were picked with 20 μl micropipette and transferred in microfuge tubes. The isolated spheres that represent each clone were dispersed into single cells with 100 μl of 0.5% Trypsin-EDTA and plated for sphere growth for 2 to 3 weeks. To ensure the purity of the clones, spheres were picked again from the growing spheres and subject to dispersion for second sphere culture (Fig. 6a).

### Genomic DNA RNA and cDNA preparation for PCR and qRT–PCR

Genomic DNA was prepared using the DNeasy Blood and Tissue Kit (Qiagen Sciences, MD). A total of 30–50 ng of genomic DNA was used per the PCR reaction. PCR amplicons were used for agarose gel electrophoresis for assessing the presence of transgenes, or purified with DNA Clean and Concentrator Kit (Zymo Research, Irvine, CA) for sequencing and subsequent TIDE analyses to assess indel efficiency^14^. Total RNA was prepared with QIAGEN RNeasy micro kit (Qiagen Sciences) or PicoPure RNA Isolation Kit (Thermo Fisher Scientific), and used for cDNA synthesis using QIAGEN Omniscript RT Kit (Qiagen), according to the manufacturer's protocol. Relative mRNA level was measured by qRT–PCR of each cDNA in duplicate with gene-specific probe sets (Applied Biosystems, Foster City, CA) with TaqMan Universal PCR Master Mix (Applied Biosystems) and the ABI Prism 7500 detection system (Applied Biosystems). Normalizations across samples were performed using β-actin primers. PCR was performed with Accuprime Pfx (Thermofisher) in the presence or absence of 4 M betaine (Sigma-Aldrich) in 35 cycles, each cycle composed of a denaturing step at 95 °C for 1 min, an annealing step at 60 °C for 1 min and an extension step at 68 °C for 1 min, followed by final extension at 68 °C for 5 min. Primer sequences are shown in Supplementary Table 7.

### Off-target effect analysis

Off-target site prediction was performed using Cas9 online designer (http://cas9.wicp.net)^38^. The potential off-target sites with the off-target scores more than 0.1 were selected, which resulted in two potential off-target sites for sgRNA CDKN2A#1, one for SMAD4#1 and no sites were detected for TP53#2. PCR primers encompassing the potential off-target sites were designed and the PCR amplicons were purified and sequenced for TIDE analyses as described above. PCR was performed with Accuprime Pfx (Thermofisher) in the presence or absence of 4 M betaine (Sigma-Aldrich) in 35 cycles, each cycle composed of a denaturing step at 95 °C for 1 min, an annealing step at 60 °C for 1 min and an extension step at 68 °C for 1 min, followed by final extension at 68 °C for 5 min. Primer sequences are shown in Supplementary Table 7.

### Orthotopic transplantation

Orthotopic transplantation was performed in the pancreas of NSG mice^39^. A total 0.75–1 × 10^6^ of dispersed single cells of human spheres or HPDE were resuspended in 100 μl of HBSS with 1% Matrigel and injected into the splenic lobe of pancreas. We estimated a minimum of three mice per each cell type for a proper evaluation of their growth as tumour or PanINs. Therefore, three to five male mice with the age of 10–14 weeks were randomized, given unique ID numbers for blinding and used per cell type for transplantation.

### Histology and immunohistochemistry

Human spheres grown in Matrigel were embedded in the histogel and fixed with 4% paraformaldehyde overnight at 4 °C, embedded in paraffin and sectioned in 5 μm thickness^40^. Organs or tumours from transplanted mice were fixed with 4% paraformaldehyde overnight at 4 °C. For frozen section, the fixed tissues were cryoprotected in 30% sucrose solution in PBS overnight, embedded in OCT on dry ice and sectioned in 10 μm thickness. For paraffin section, the fixed histogel block or tissues were dehydrated with a series of ethanol solutions and xylene, embedded in paraffin and sectioned in 5 μm thickness. Haematoxylin and eosin staining, Alcian blue staining (NovaUltra Alcian Blue Stain Kit; IHC World, Ellicott City, MD) and immunohistochemistry were performed with either frozen or paraffin sections. The primary antibodies used were rabbit anti-phospho-AKT (clone 736E11; 1:50; Cell Signaling Technology, Danvers, MA), mouse anti-CD133 (clone AC133 and 293C3; 1:100 each; Miltenyi Biotec, Auburn, CA), rabbit anti-CK19 (319R-15; 1:200; Cell Marque, Rocklin, CA), rabbit anti-phospho-ERK (clone D13.14.4E; 1:500; Cell Signaling Technology), rat anti-F4/80 (MCA497RT; 1:500; Bio-Rad, Hercules, CA), mouse anti-HuNu (MAB1281; 1:200; Millipore, Billerica, MA), rabbit anti-Ki-67 (NCL-Ki67p; 1:100; Leica Microsystems, Germany), rat anti-Ly-6B.2 (MCA771GT; 1:500; Bio-Rad, Hercules, CA), rat anti-MAC-2 (CL8942AP; 1:1,000; Cedarlane, Burlington, NC), mouse anti-mitochondria (AB92824, 1:1,000; Abcam, Cambridge, MA), mouse anti-MUC5AC (MRQ-19; 1:100; Cell Marque), mouse anti-NMU (clone 2A16; Kim Laboratory) and rabbit anti-phospho-STAT3 (clone D3A7; 1:500; Cell Signaling Technology). Antigen unmasking with Target Retrieval Solution (Dako, Carpinteria, CA) was used for anti-F4/80, Ly-6B.2, MAC-2 and MUC5AC antibodies; 10 mM sodium citrate buffer for anti-human mitochondria and phospho-ERK antibodies; and TRIS/EDTA (pH 9.0) buffer for anti-phospho-AKT, NMU and phospho-STAT3 antibodies. Secondary antibodies used were from Jackson ImmunoResearch (West Grove, PA) or Molecular Probes (Eugene, OR). Stained sections were mounted with VECTASHIELD Mounting Medium with DAPI (Vector Laboratories) or Permount (Thermo Fisher Scientific). Fluorescence or colour images were taken using Zeiss Axio Imager.M2 or Leica SP2 inverted confocal laser scanning microscope. All our histological data were evaluated independently by two pathologists (R.H.H. and T.A.L.), based on both morphological and cytological features of the lesions.

### RNA isolation and RNA-Seq assays

RNA quality and quantity was measured using Bioanalyser instrument (Agilent Technologies). RNA-Seq libraries were built using KAPA Stranded mRNA-Seq Kit (KK8400, KAPA Biosystems, Wilmington, MA). Barcoded libraries were multiplexed and sequenced as single-end 75 bp reads on Illumina NextSeq 500 sequencer. RNA-Seq data sets were aligned to the Human Genome Assembly hg19 and analysed using DNAstar ArrarStar and Qseq version 12.3.1 build 53 (DNASTAR Inc., Madison, WI) with default parameters. Only the genes with more than 1 average RPKM (reads per kilobase million) value of all clones were used for fold change and GSEA analyses. For GSEA, GSEA v2.2.2 Software (Broad Institute) was used with Hallmark gene sets (H) in the Molecular Signatures Database (MSigDB) v5.1. Custom human PanIN and PDA genesets were generated by downloading publically available expression data sets that compare human PanIN or PDA with normal pancreas or normal ducts from Pancreatic Expression Database^41^. Due to minimum geneset size limit (16 or more genes per geneset), total 34 genesets with geneset size ranging from 16 to 334 were generated from 20 PanIN and PDA expression studies. The custom human PanIN and PDA genesets are available on request. In all cases, only the signatures showing normalized enrichment score≥1.5, *P* value<0.01 and false discovery rate *q*-value <0.05 were considered significant.

### Study approval

Institutional review board approval for research use of human tissue was obtained from the Stanford University School of Medicine and Johns Hopkins School of Medicine. Written informed consent was received for all human tissues used in this study. All animal experiments and methods were approved by the Institutional Animal Care and Use Committee of Stanford University.

### Data availability

All sequencing data that support the findings of this study have been deposited in the National Center for Biotechnology Information Gene Expression Omnibus (GEO) and are accessible through the GEO Series accession number GSE88997. All other relevant data are available from the corresponding author on request.

## Additional information

**How to cite this article:** Lee, J. *et al*. Reconstituting development of pancreatic intraepithelial neoplasia from primary human pancreas duct cells. *Nat. Commun.* **8,** 14686 doi: 10.1038/ncomms14686 (2017).

**Publisher's note:** Springer Nature remains neutral with regard to jurisdictional claims in published maps and institutional affiliations.

## Supplementary Material

Supplementary Information Supplementary Figures, Supplementary Tables, Supplementary Notes.

Supplementary Data 1 RNAseq analysis result with fold changes and a list of reported genes differentially expressed in PanIN or PDA.

## Figures and Tables

**Table 1:** List of PanIN-like structures found in each mouse pancreas transplanted with sphere clones.

**Sample ID**	**Mouse ID**	**Human graft found**	**Pathology review**
S1 KCST^CLONE3^	199	Yes	PanIN2
	200	No	N/A
	201	No	N/A
			
S1 KCST^CLONE4^	202	No	N/A
	203	Yes	PanIN2
	204	Yes	PanIN2, PanIN3
	206	Yes	PanIN2
	207	Yes	PanIN3
			
S1 KECST^CLONE4^	197	Yes	PanIN2
	208	No	N/A
	209	Yes	PanIN2, PanIN3
			
S2 KCT^CLONE3^	195	No	N/A
	196	No	N/A
	232	No	N/A
	233	Yes	Normal duct
	234	Yes	Normal duct
			
S2 KECST^CLONE8^	261	Yes	PanIN1
	262	Yes	PanIN2
	263	Yes	PanIN1, PanIN2

N/A, not applicable.
