# Supplementary material for: Reconstituting development of pancreatic intraepithelial neoplasia from primary human pancreas duct cells
Source: Nat Commun. 2017 Mar 8;8:14686. doi: 10.1038/ncomms14686 (PMC5344977; doi:10.1038/ncomms14686)

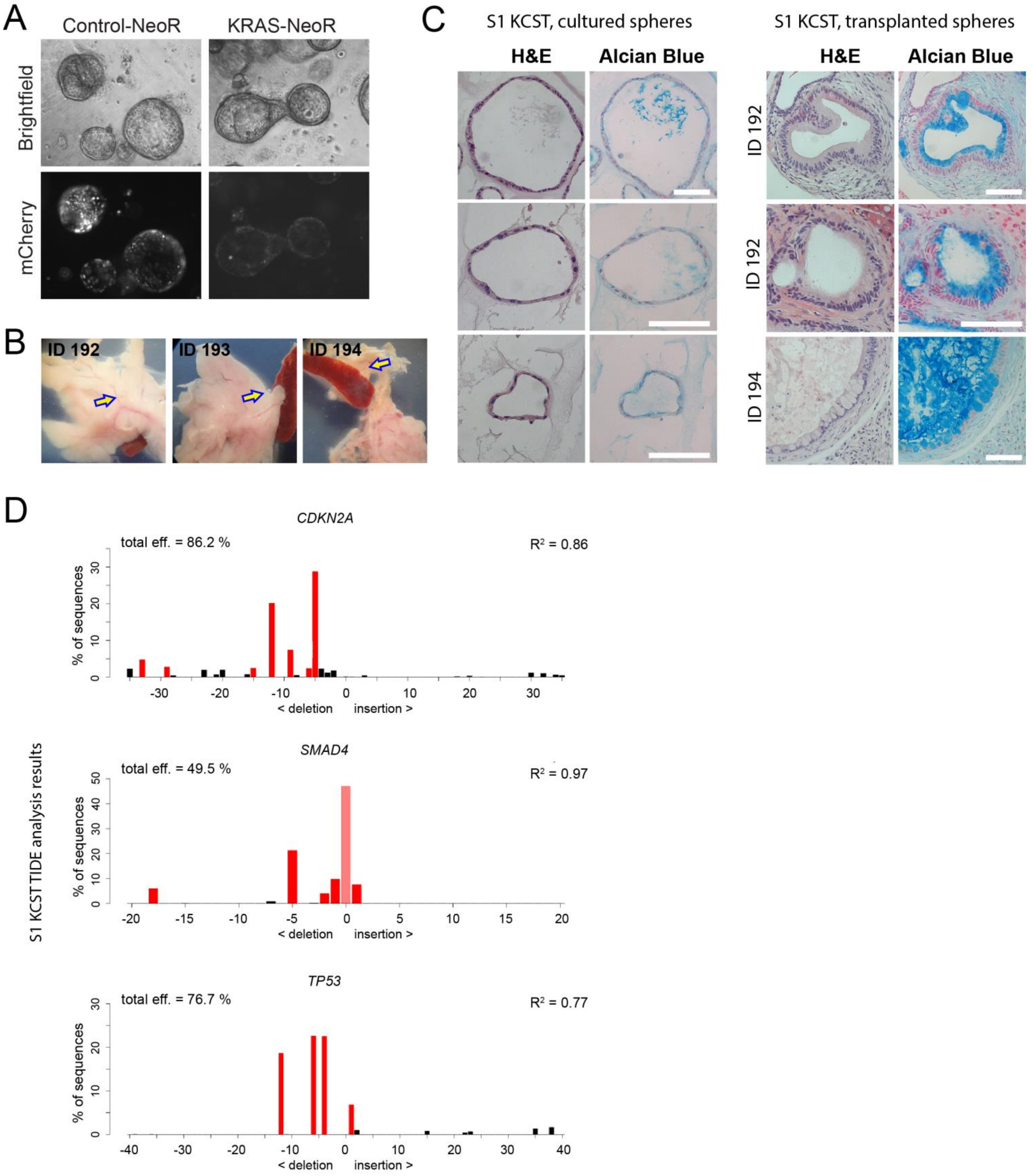

Supplementary Figure 1. Phenotype and genotype of cultured and transplanted S1 KCST

(A) Brightfield and mCherry fluorescence images of the spheres generated from the CD133-positive cells infected with either Control-NeoR or KRAS-NeoR. (B) Transplanted pancreas with S1 KCST spheres. Yellow arrows indicate the cyst-like structures found during pancreas dissection. (C) Representative images of H&E and Alcian blue staining from S1 KCST cultured spheres (left) and transplanted mice (right). Note that no alcian blue-positive cytoplasm in cultured spheres. Scale Bars, 100μM. (D) Indel spectrum images of TIDE analysis for S1 KCST spheres. Red and black bars show $p < 0.05$ and $p > 0.05$, respectively. Orange bar shows the peak with no Indel mutations.

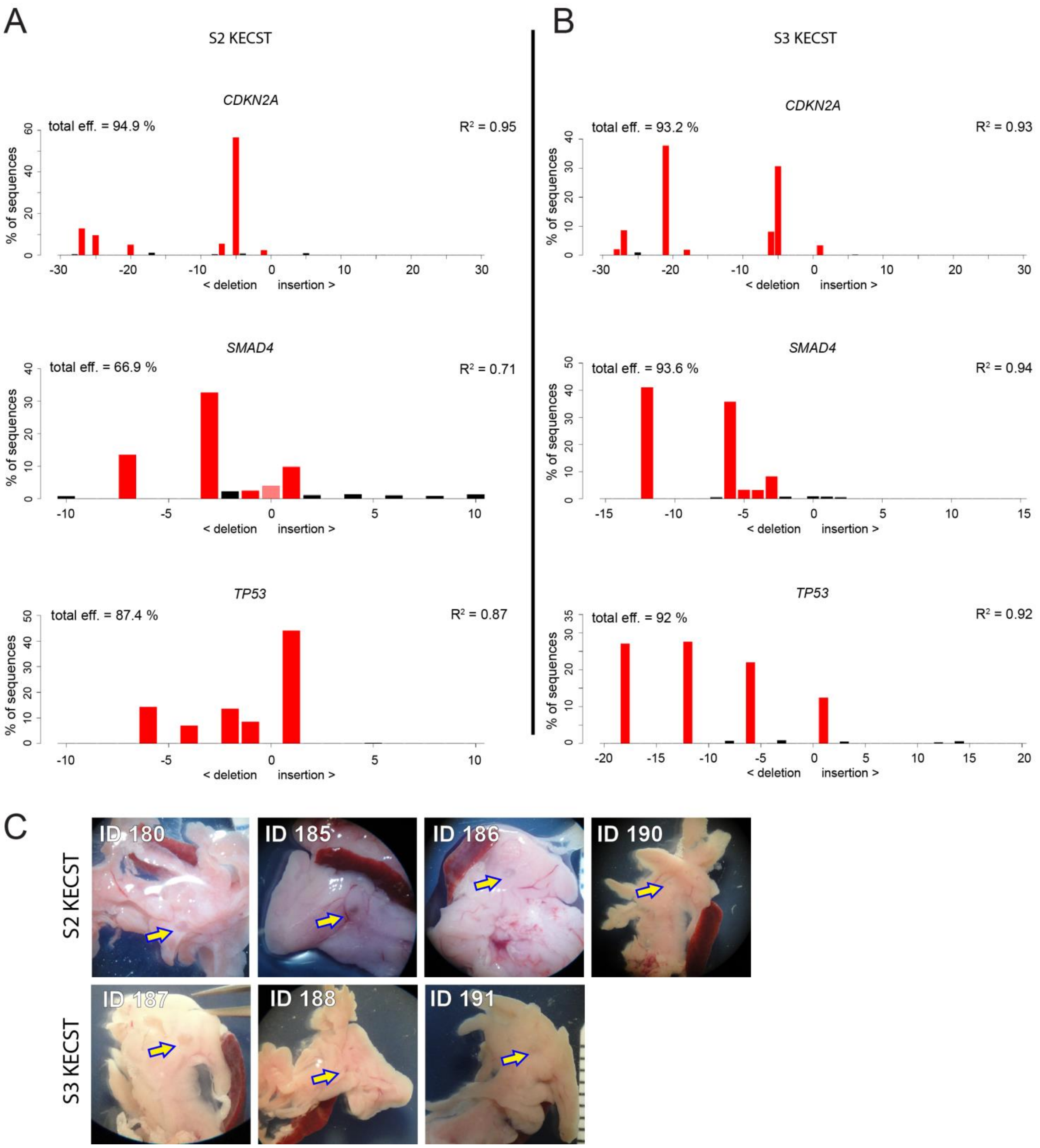

Supplementary Figure 2. Phenotype and genotype of cultured and transplanted S2 and S3 spheres

(A and B) Indel spectrum images of TIDE analysis for S2 KECST (A) and S3 KECST (B) spheres. (C) Transplanted pancreas with S2 and S3 KECST spheres. Yellow arrows indicate the cyst-like structures found during pancreas dissection.

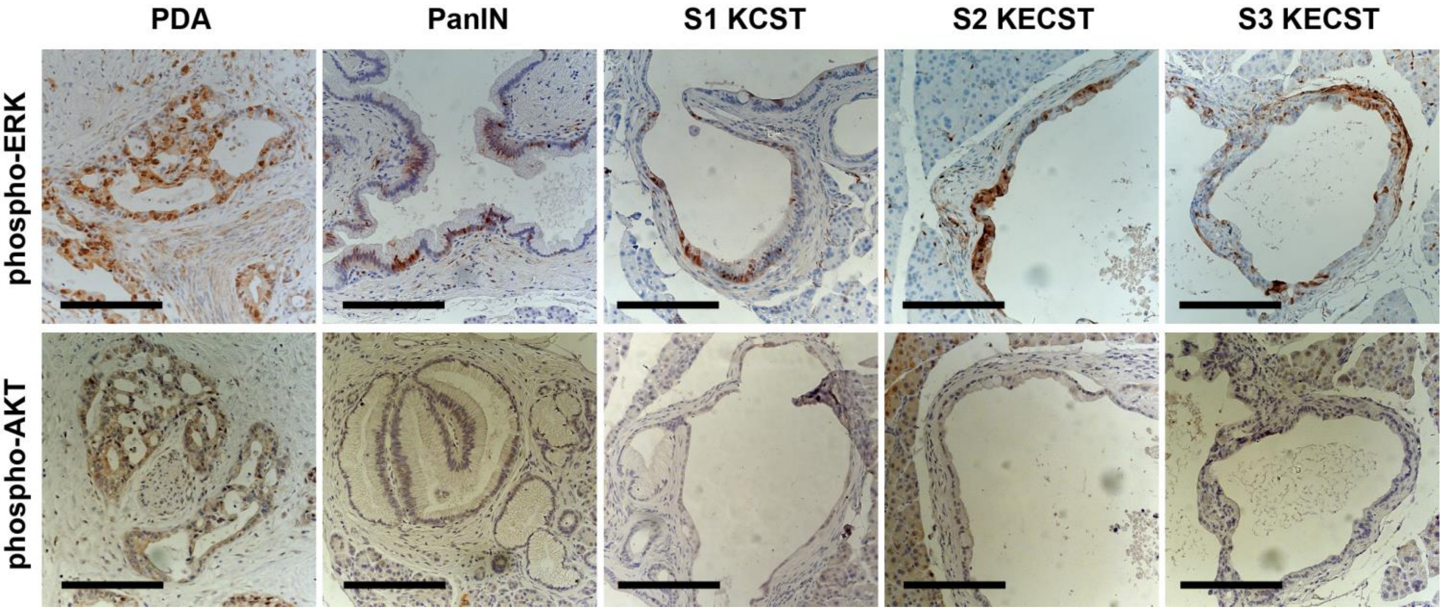

Supplementary Figure 3. Immunohistochemistry with antibodies against phospho-ERK and phospho-AKT
Human PDA, PanIN, and pancreas transplanted with S1 KCST, S2 KECST, and S3 KECST spheres was stained with anti-phospho-ERK and phospho-AKT antibodies. Note that phospho-AKT signal is weak or absent in human native PanIN and all transplanted hiPanIN lesions. Scale Bars, 200μM.

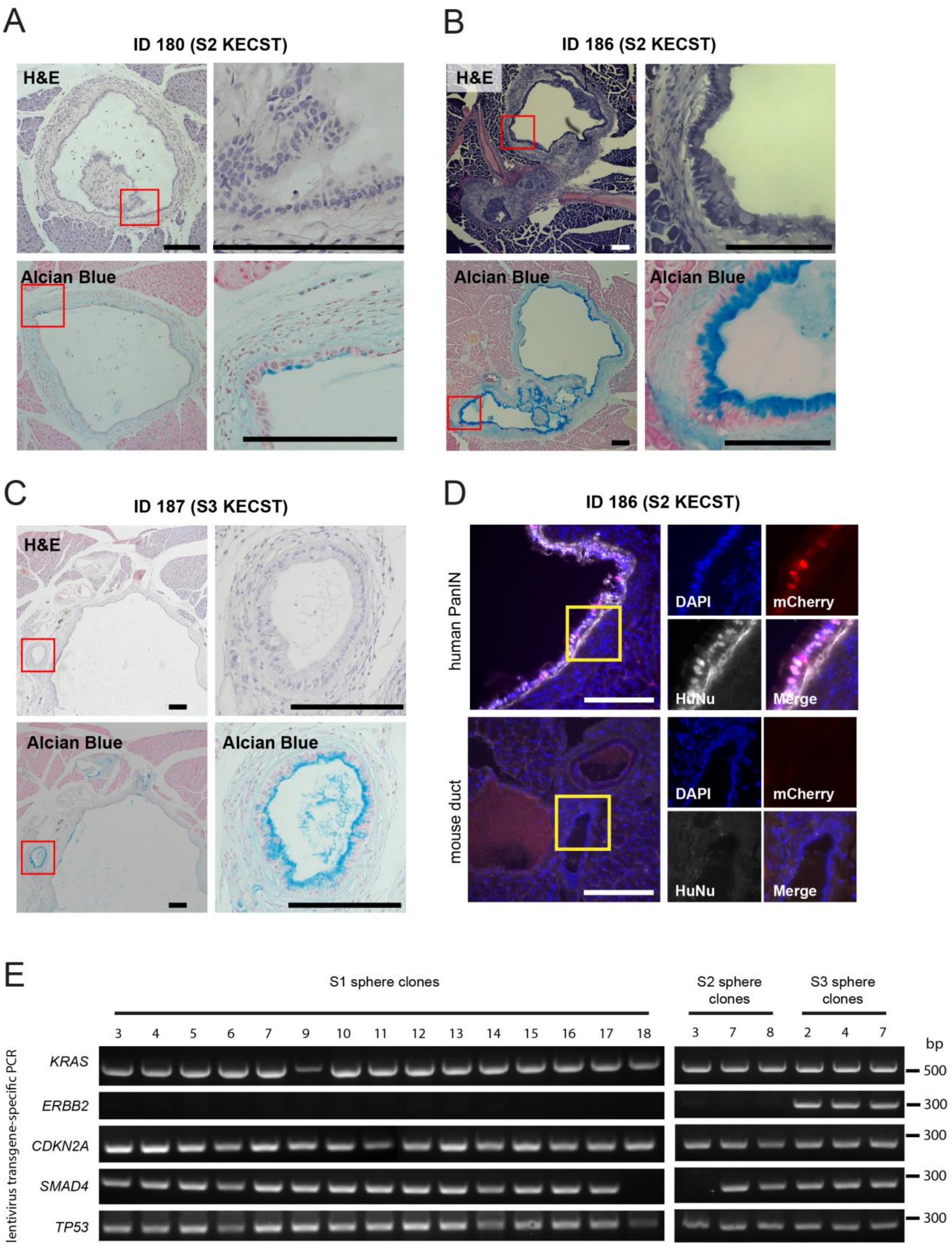

Supplementary Figure 4. Phenotype and genotype of cultured and transplanted spheres
(A-C) H&E and Alcian blue staining of the transplanted S2 KECST (A and B) and S3 KECST (B) spheres. (D) Pancreas transplanted with S2 KECST spheres was stained with human nuclear-specific antibody (HuNu, white) and mCherry fluorescence (red) along with DAPI nuclear staining (blue). Note that neither mCherry- nor HuNu-positive cells were found in mouse duct of the same pancreas. Magnified view of the boxed areas are shown to the right. Scale Bars, 200μM. (E) Genomic DNA PCR of cloned spheres for assessing the presence of lentiviral transgenes. Numbers on top denote clone numbers.

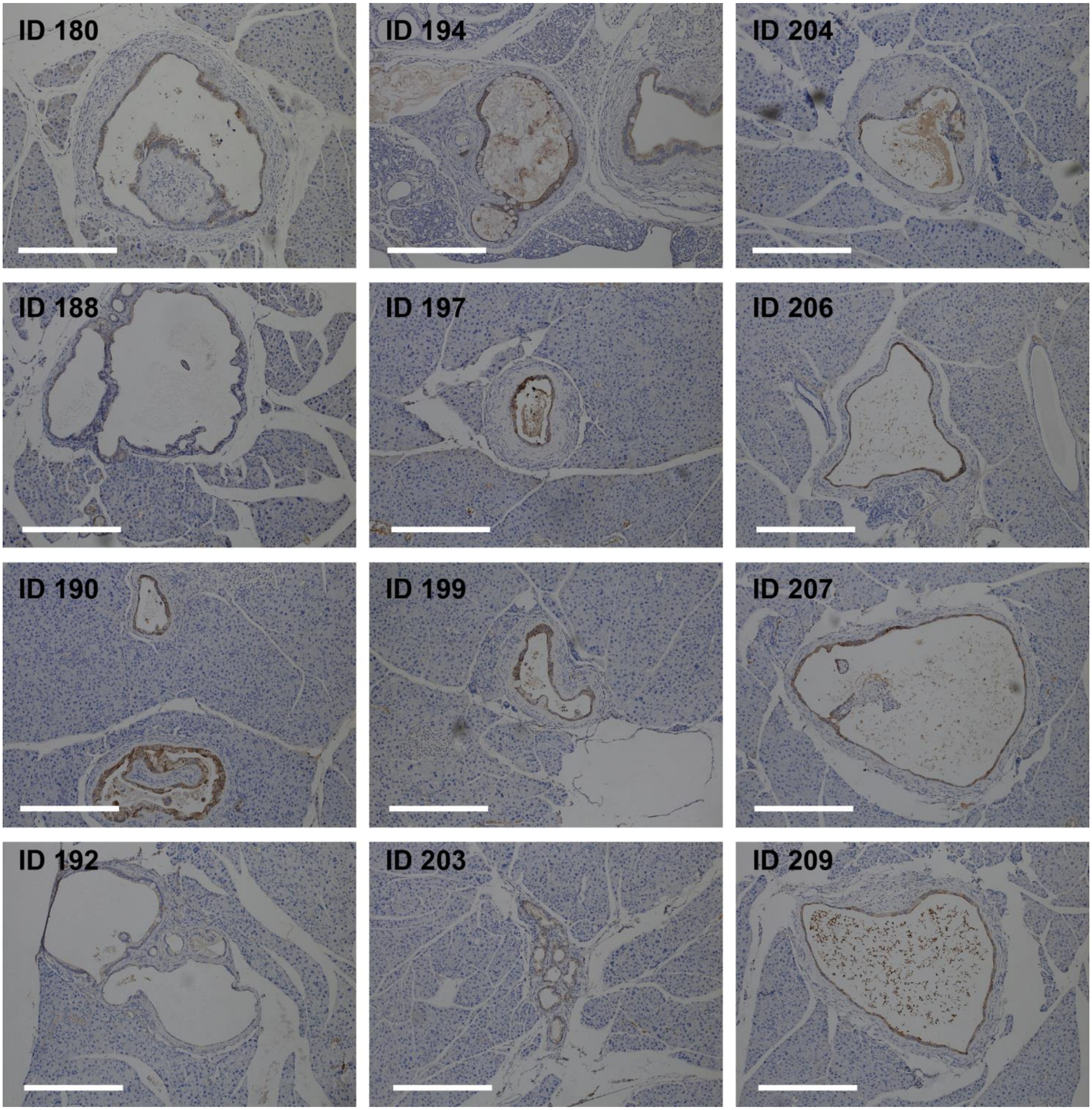

Supplementary Figure 5. Immunohistochemistry with anti-human mitochondria antibody
Pancreas transplanted with S1 KCST, S2 KECST, and S3 KECST spheres was stained with anti-human mitochondria antibody. Scale Bars, 200µM.

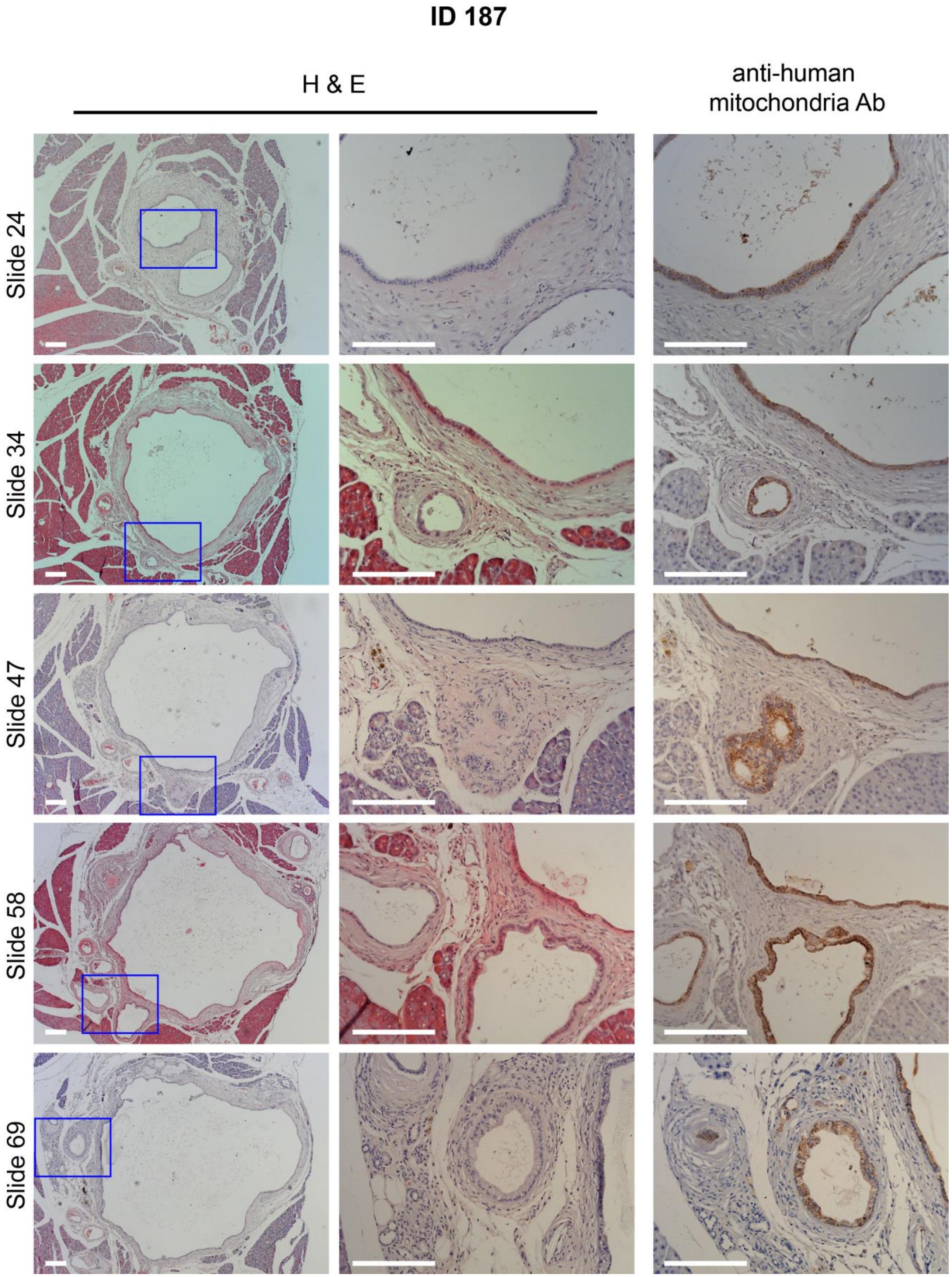

Supplementary Figure 6. Hematoxylin and Eosin and anti-human mitochondria antibody staining on serial sections
Serial sections of the pancreas transplanted with S2 KECST (ID 187) spheres were stained with H&E and anti-human mitochondria antibody. Scale Bars, 200µM.

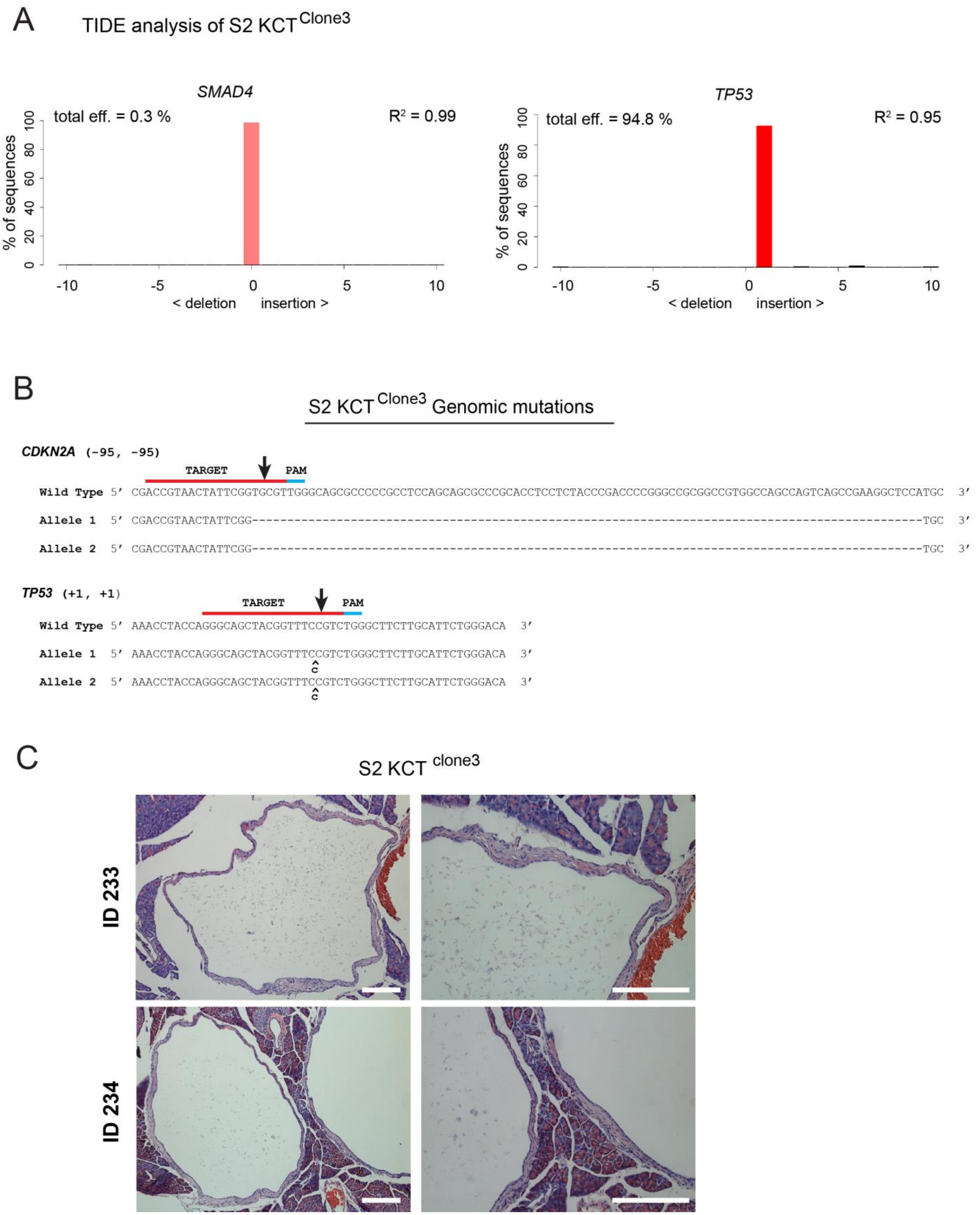

Supplementary Figure 7. Genotype and phenotype of S2 KCT^{clone3}

(A) TIDE Indel spectrum images of TIDE analysis for S2 KCT^{clone3}. Note that Indel spectrum for CDKN2A is not available due to the large size of deletion. (B) Genomic DNA sequences of CRISPR-Cas9-targeted loci. The numbers next to the gene names indicate the total number of nucleotide deleted or inserted. Arrows indicate the expected cut site by Cas9 nuclease. (C) H&E staining of the human grafts found in transplanted pancreas with S2 KCT^{clone3}. Scale Bars, 200µM.

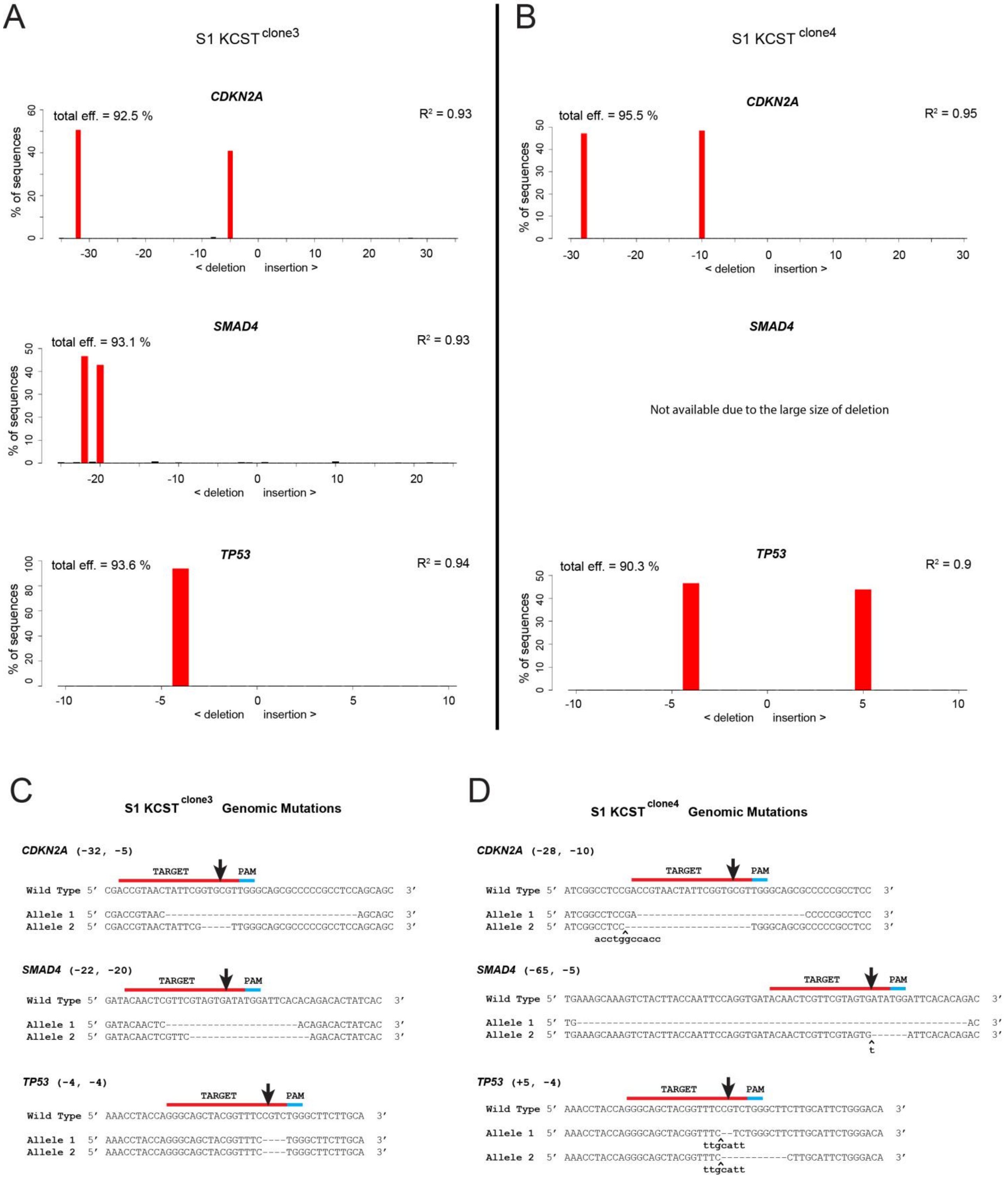

Supplementary Figure 8. Genotype and phenotype of S1 clones

(A and B) TIDE Indel spectrum images of TIDE analysis for S1 KCST^{clone3} (A) and S1 KCST^{clone3} (B). (C and D) Genomic DNA sequences of CRISPR-Cas9-targeted loci for S1 KCST^{clone3} (C) and S1 KCST^{clone3} (D). The numbers next to the gene names indicate the total number of nucleotide deleted or inserted. Arrows indicate the expected cut site by Cas9 nuclease.

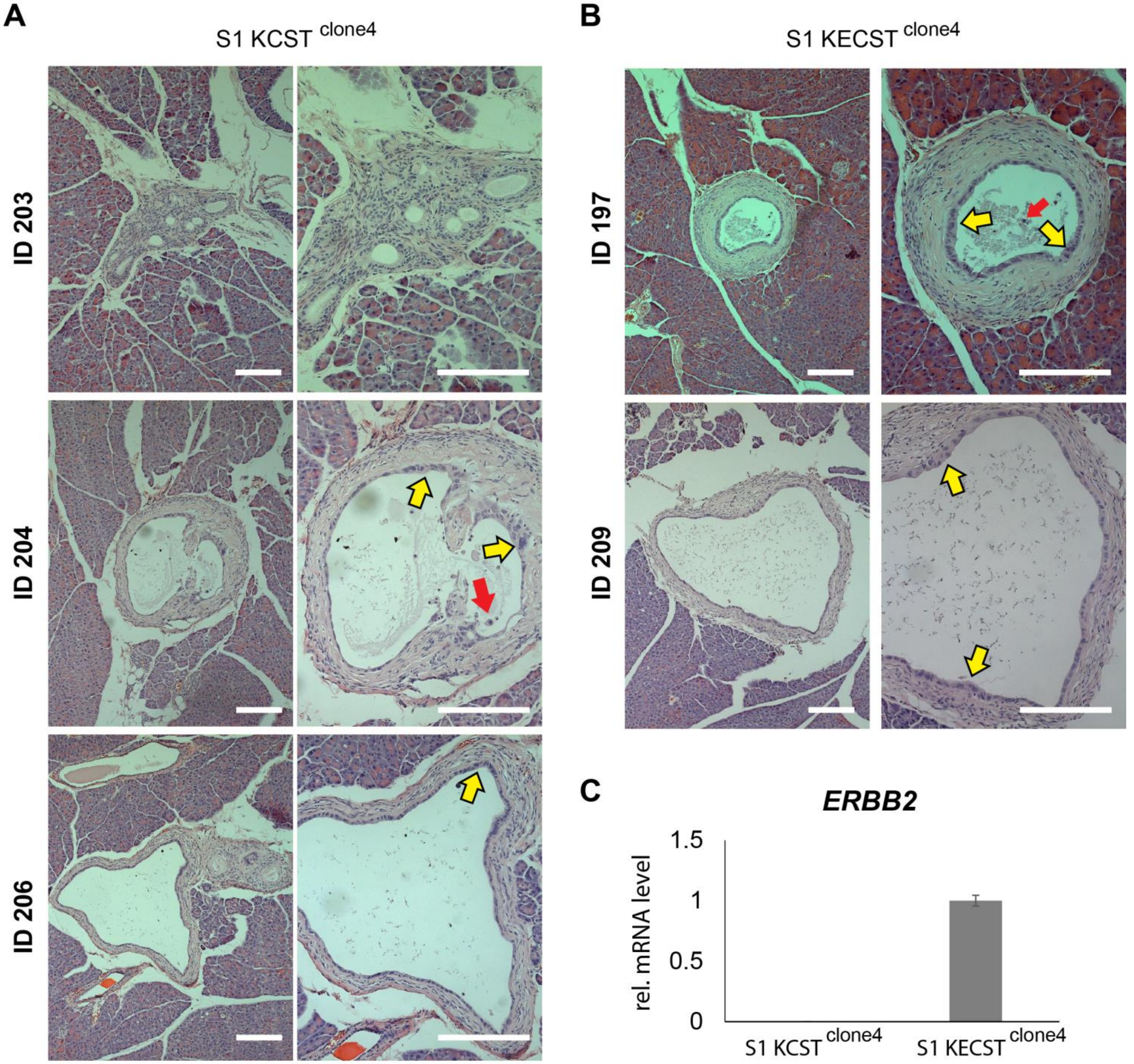

Supplementary Figure 9. H&E images of S1 clones

(A-B) H&E staining of PanIN structures found in transplanted pancreas with S1 KCST^{clone4}. (A) and S1 KECST^{clone4} (B). Note that the PanIN structures show cribriforming (A, ID203), abnormal nuclei (yellow arrows), and necrotic cells in the lumen (red arrows), features of human PanIN2 and 3. Magnified images are shown on right. (C) Relative mRNA expression level of ERBB2 transgene Error bars = S.D. n = 2. Scale Bars, 200µM.

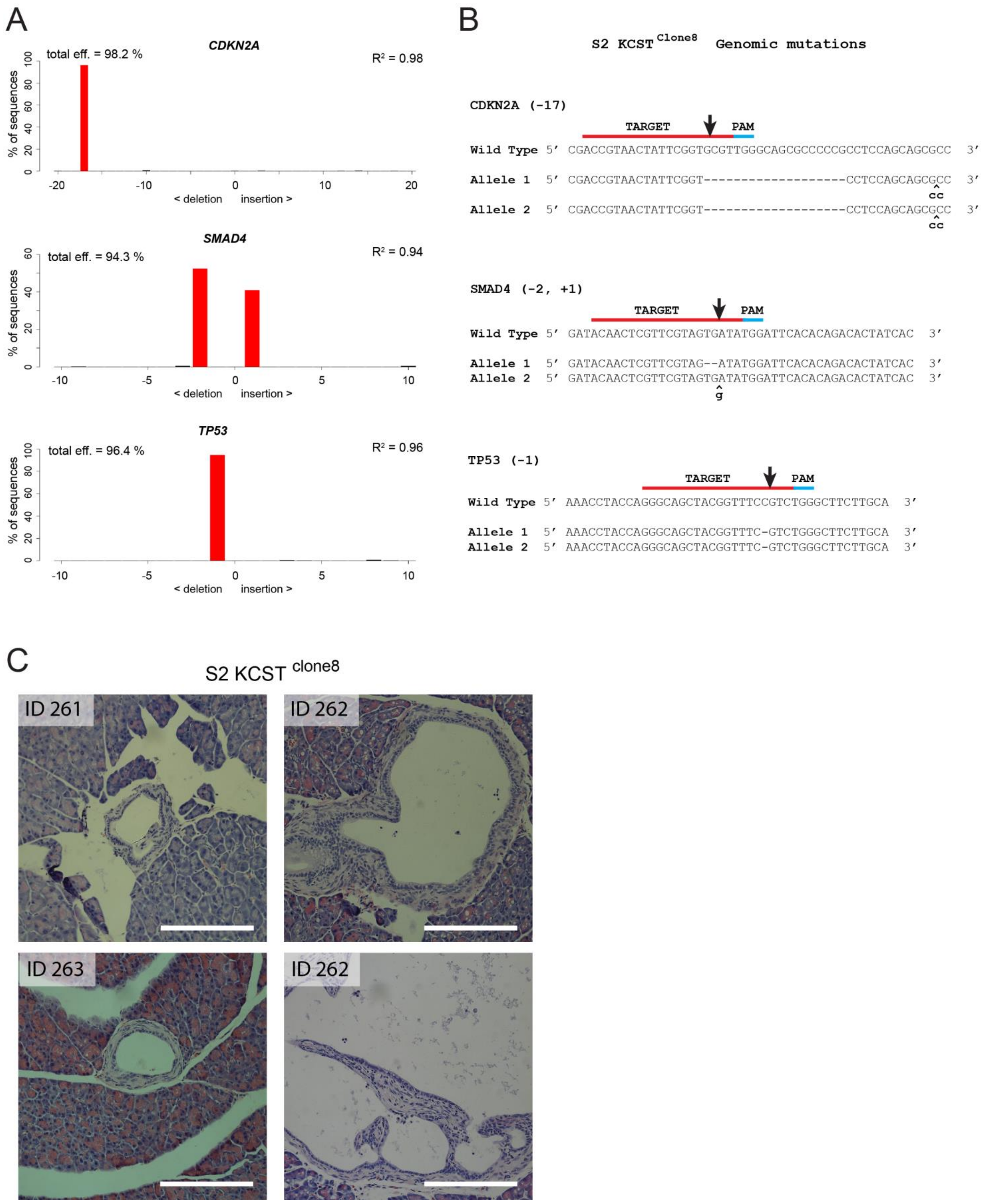

Supplementary Figure 10. Genotype and phenotype of S2 KCST^{clone8}

(A) TIDE Indel spectrum images of TIDE analysis for S2 KCST^{clone8}. (B) Genomic DNA sequences of CRISPR-Cas9-targeted loci for S2 KCST^{clone8}. The numbers next to the gene names indicate the total number of nucleotide deleted or inserted. Arrows indicate the expected cut site by Cas9 nuclease. (C) H&E staining of PanIN structures found in transplanted pancreas with S2 KCST^{clone8}. Scale Bars, 200µM.

S1 clone3 KCST

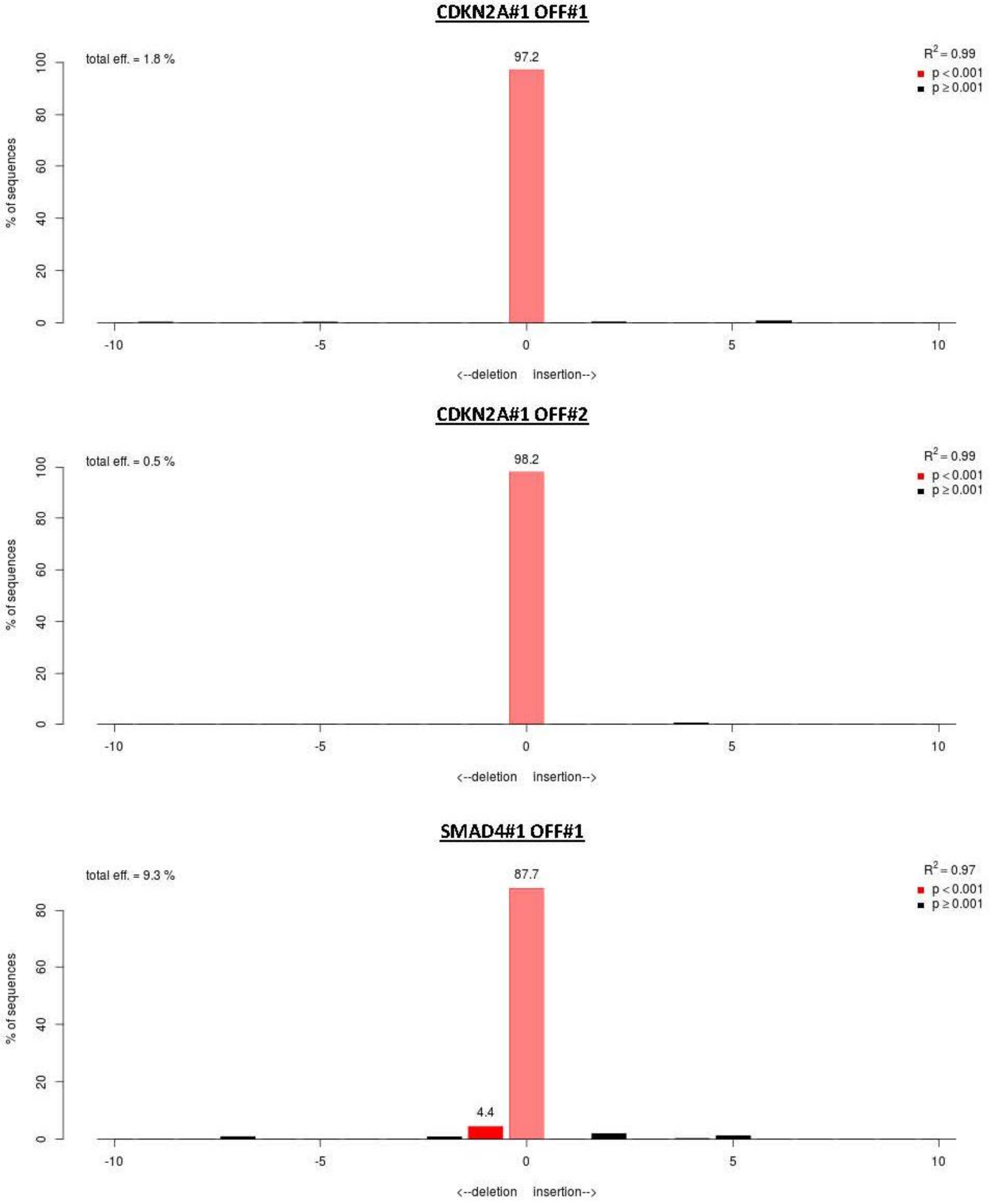

Supplementary Figure 11. Off-target analysis result of S1 KCST^{clone3}
Off-target analysis result of S1 KCST^{clone3}. See Supplementary Table 4.

S1 clone4 KCST

CDKN2A#1 OFF#1

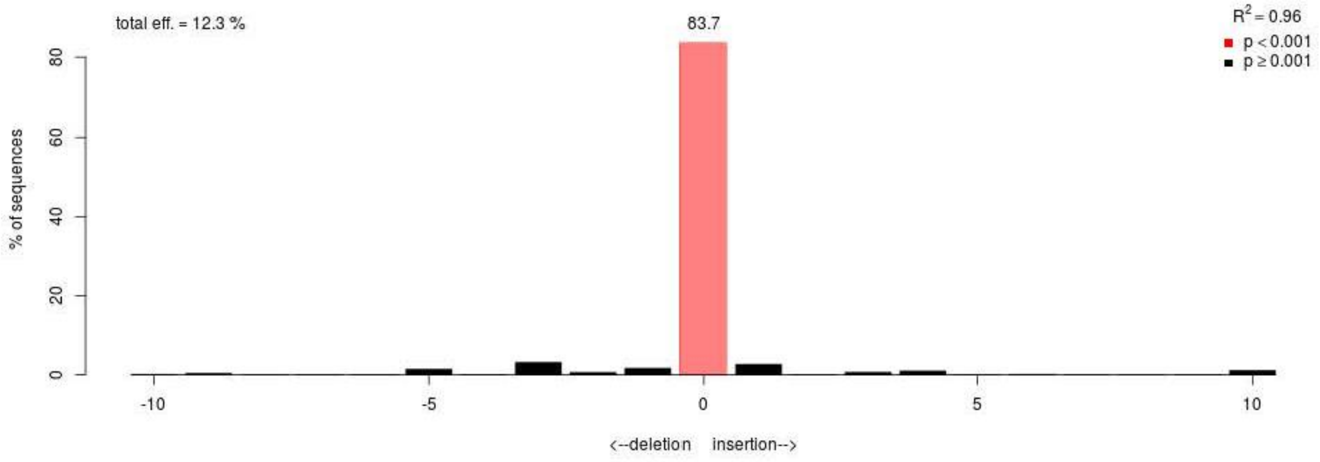

CDKN2A#1 OFF#2

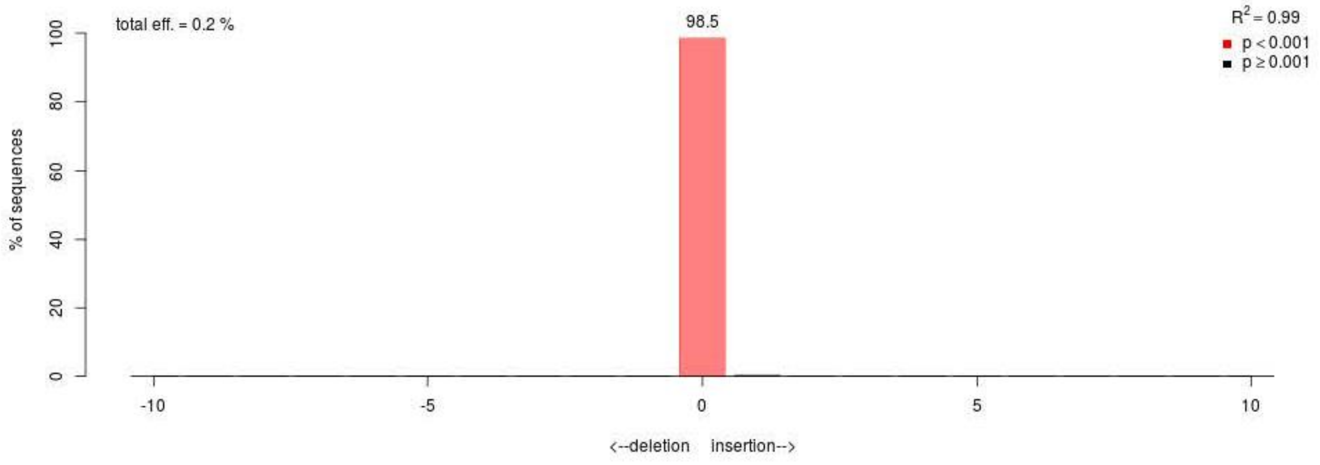

SMAD4#1 OFF#1

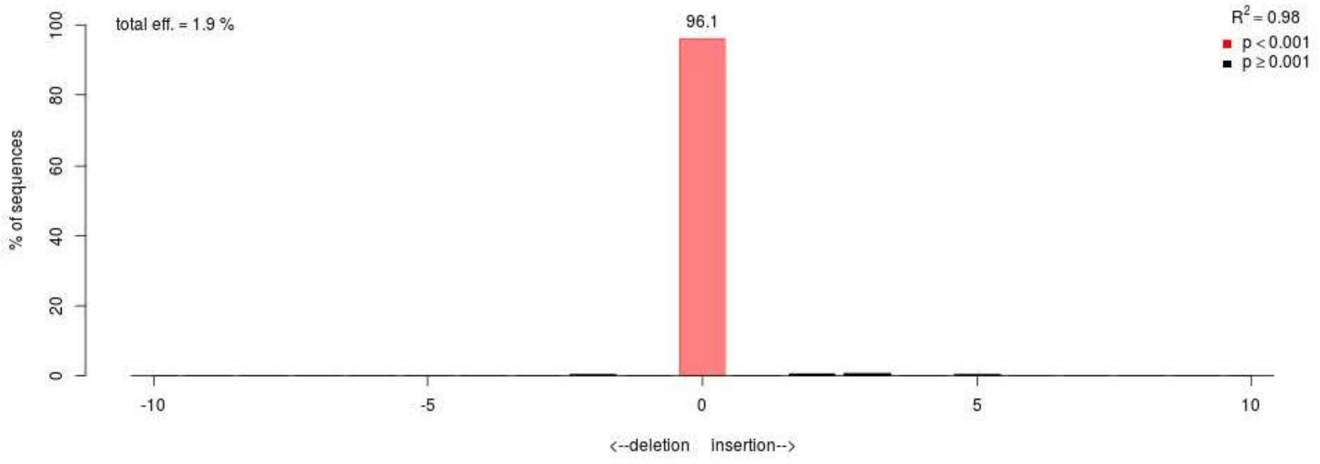

Supplementary Figure 12. Off-target analysis result of S1 KCST^{clone4}
Off-target analysis result of S1 KCST^{clone4}. See Supplementary Table 4.

S2 clone3 KCT

CDKN2A#1 OFF#1

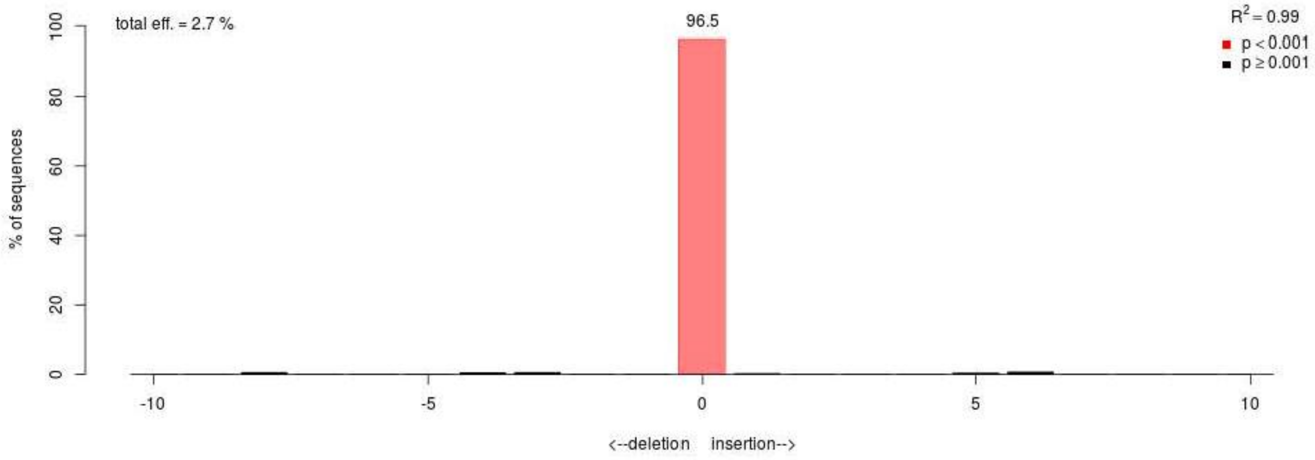

CDKN2A#1 OFF#2

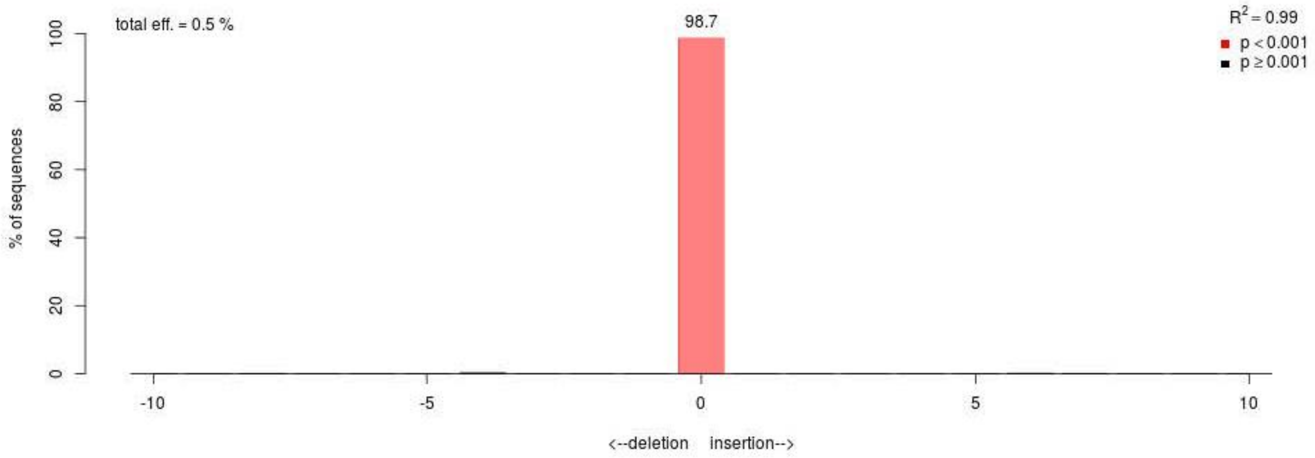

SMAD4#1 OFF#1

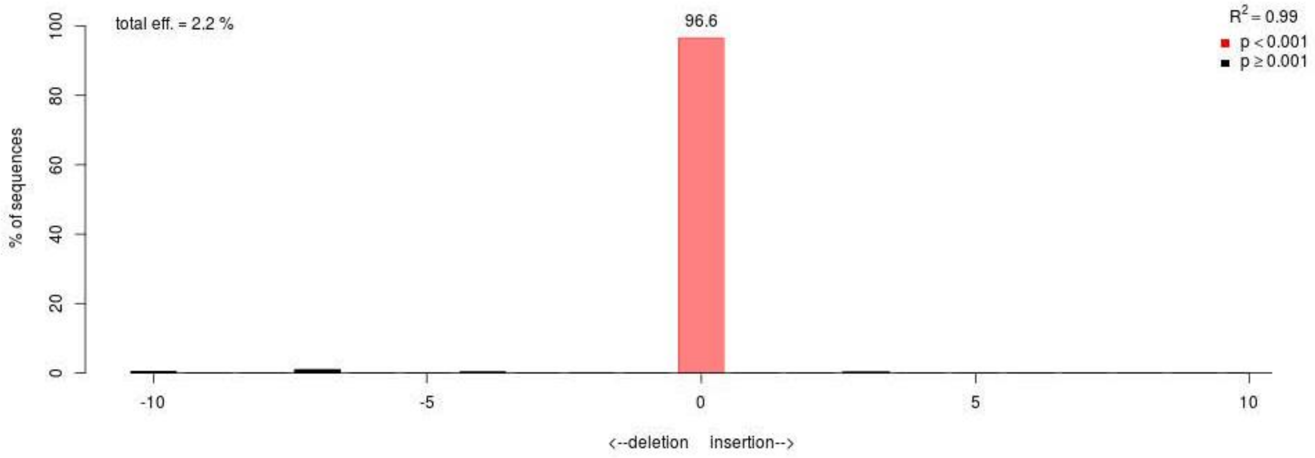

Supplementary Figure 13. Off-target analysis result of S2 KCT^{clone3}
Off-target analysis result of S2 KCT^{clone3}. See Supplementary Table 4.

S2 clone8 KCST

CDKN2A#1 OFF#1

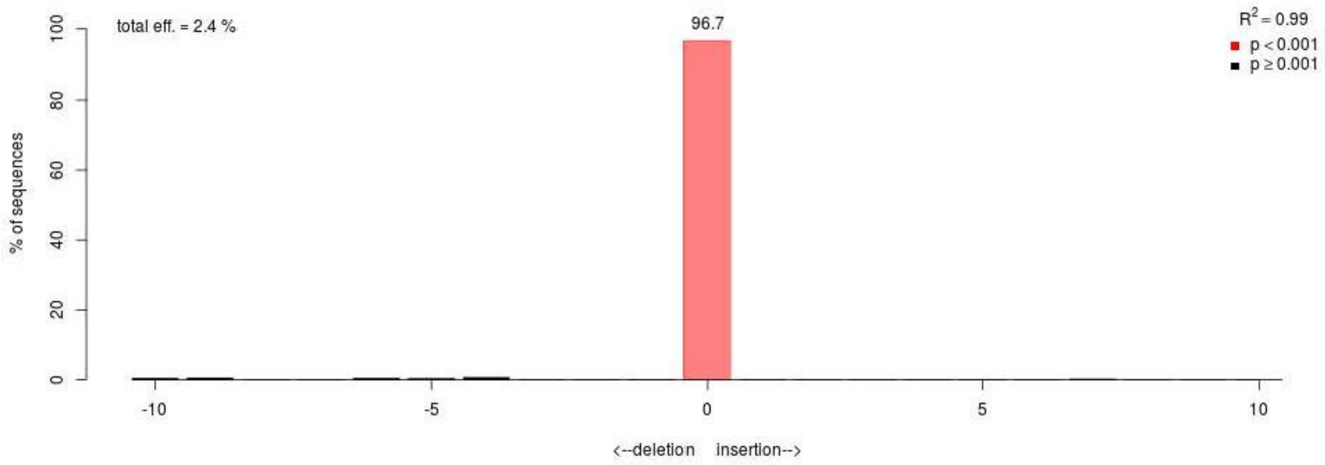

CDKN2A#1 OFF#2

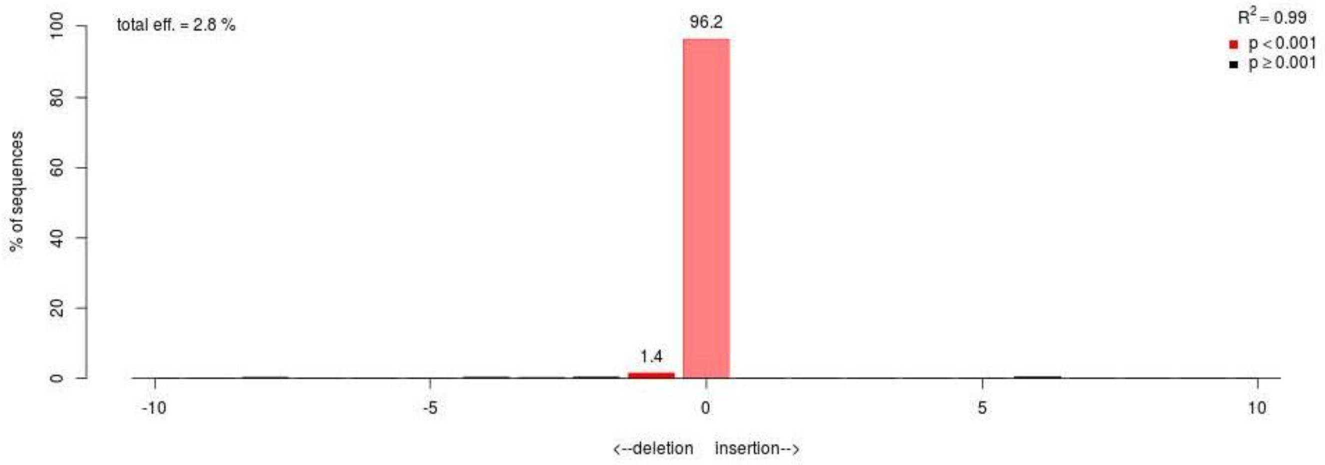

SMAD4#1 OFF#1

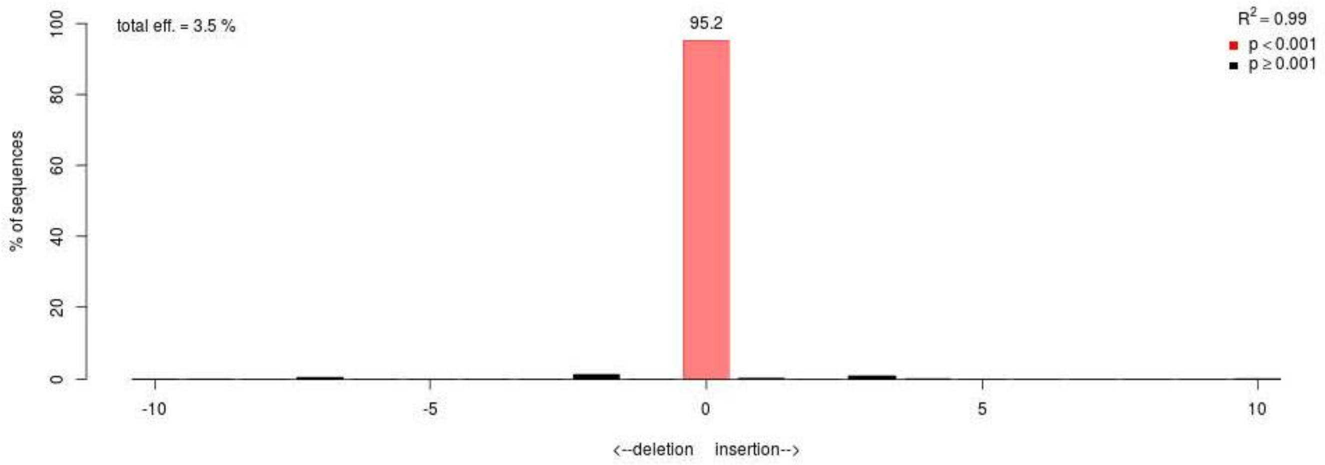

Supplementary Figure 14. Off-target analysis result of S2 KCST^{clone8}
Off-target analysis result of S2 KCST^{clone8}. See Supplementary Table 4.

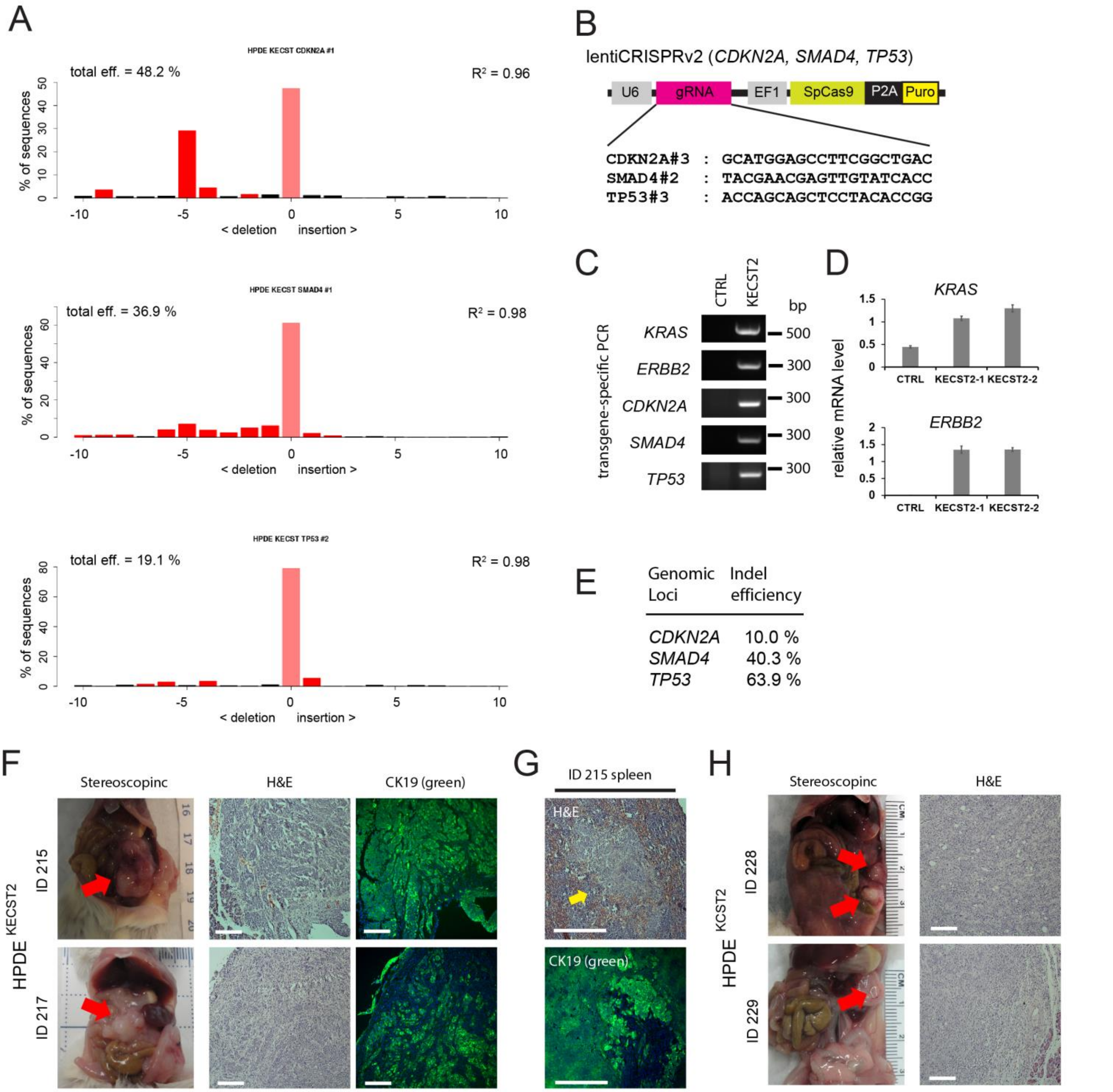

Supplementary Figure 15. Genetic modification of human ductal cell line HPDE induces invasive PDA development
(A) Indel spectrum images of TIDE analysis for $\text{HPDE}^{\text{KECST}}$ (B) Schematics of lentiCRISPRv2 and new sets of sgRNA sequences (KECST2). (C) Genomic DNA PCR for assessing the presence of lentiviral transgenes in HPDE cells. (D) Relative mRNA expression level of oncogenic *KRAS* and *ERBB2* transgene. Error bars = S.D. (E) Indel efficiency of each genomic loci assessed by TIDE analysis. (F) Stereoscopic and representative H&E and CK19 (green) staining images of the tumors formed in the transplanted pancreas with $\text{HPDE}^{\text{KECST2}}$. Red arrows indicate tumor nodules. (G) Representative H&E staining of spleen with metastatic cells found in ID 215. The metastatic cells are CK19-positive (green, bottom). (H) Stereoscopic and representative H&E staining images of the tumors formed in the transplanted pancreas with $\text{HPDE}^{\text{KCST2}}$. Scale Bars, 200μM.

Supplementary Table 1. Phenotypes of pancreas donors

ANONYMOUS ID	AGE (YEAR)	GENDER	BODY MASS INDEX
**S1**	54	M	40.3
**S2**	40	F	25.2
**S3**	51	M	28.4

Supplementary Table 2. List of PanIN-like structures in transplanted mouse pancreas

SAMPLE ID	MOUSE ID	HUMAN GRAFT FOUND	PATHOLOGY REVIEW
**S1 KCST**	192	YES	PanIN1
	193	YES	PanIN1
	194	YES	PanIN1
**S2 KECST**	179	YES	PanIN1, PanIN2
	180	YES	PanIN2
	181	NO	N/A
	185	YES	PanIN1
	186	YES	PanIN1
	190	YES	PanIN2
**S3 KECST**	187	YES	PanIN1
	188	YES	PanIN1, PanIN2
	191	YES	PanIN1

Supplementary Table 3. List of isolated sphere clones and their genotypes

Sample ID	Clone ID	Presence of Transgenes					Indel mutation status			Note
		K	E	C	S	T	CDKN2A	SMAD4	TP53	
S1 KCST	3	Y	N	Y	Y	Y	-32/-5	-22/-20	-4/-4	S1 KCST^{clone3}
	4	Y	N	Y	Y	Y	-28/-10	-65/-5	-4/5	S1 KCST^{clone4}
	5	Y	N	Y	Y	Y	-22/-22	-2/-5	-6/-85	
	6	Y	N	Y	Y	Y	-12/-10	-24/-2	-21/-21	
	7	Y	N	Y	Y	Y	-9/-9	-5/-1	-12/-6	
	9	Y	N	Y	Y	Y	-29/-5	-5/-1	-12/-6	
	10	Y	N	Y	Y	Y	-12/-5	-5/-1	-12/-6	
	11	Y	N	Y	Y	Y	-12/-76	-5/-5	-6/-4	
	12	Y	N	Y	Y	Y	-35/-35	-5/-5	-12/1	
	13	Y	N	Y	Y	Y	N/D	-5/-5	-6/-4	
	14	Y	N	Y	Y	Y	-5/-4	0/-13	-11/4	
	15	Y	N	Y	Y	Y	N/D	-5/-5	-6/-4	
	16	Y	N	Y	Y	Y	N/D	-5/-5	-6/-4	
	17	Y	N	Y	Y	Y	-36/-5	N/D	N/D	
	18	Y	N	Y	N	Y	-21/-28	0/0	-12/4	
S2 KECST	3	Y	N	Y	N	Y	-95/-95	0/0	+1/+1	S2 KCT^{clone3}
	7	Y	N	Y	Y	Y	-25/-5	-2/-2	N/D	
	8	Y	N	Y	Y	Y	-17/-17	-2/+1	-1/-1	S2 KCST^{clone8}
S3 KECST	2	Y	Y	Y	Y	Y	-21/-21	-6/-6	-6/-6	
	4	Y	Y	Y	Y	Y	-21/-21	-6/-6	-6/-6	
	7	Y	Y	Y	Y	Y	-21/-21	-6/-6	-6/-6	

Supplementary Table 4. Off-target analysis result

sgRNA	human chromosome	alignment(dots are matched bp)	off-target score	Note
CDKN2A#1	Query_1	ACCGTAACTATTCGGTGCGTNGG	activity=1 when PAM=NGG	
	Ch9:21974692-21974714	....................T..	1	CDKN2A locus
	Ch7:153288832-153288851	....A..........T.T..	0.113715	CDKN2A#1 OFF1
	Query_1	ACCGTAACTATTCGGTGCGTNaG	activity=0.4 when PAM=NAG	
	Ch2:204638735-204638720	.............A..	0.10188864	CDKN2A#1 OFF2
SMAD4#1	Query_1	ACAACTCGTTCGTAGTGATANGG	activity=1 when PAM=NGG	
	Ch18:48575216-48575194	....................T..	1	SMAD4 locus
	Ch7:68734529-68734509	.....T..T.........G..	0.442225	SMAD4#1 OFF1
TP53#2	Query_1	GGGCAGCTACGGTTTCCGTCNGG	activity=1 when PAM=NGG	
	Ch17:7579375-7579353	....................T..	1	TP53 locus

Supplementary Table 5. Tumors found in mouse pancreas transplanted with transduced HPDE cells

SAMPLE ID	MOUSE ID	METASTASIS	PATHOLOGY REVIEW
**HPDE** ^{KECST}	211	Lung	poorly differentiated carcinoma
	212		mid-to-poorly differentiated adenocarcinoma
	214		poorly differentiated adenocarcinoma
**HPDE** ^{KCST}	219		poorly differentiated adenocarcinoma
	220		moderate-to poorly differentiated adenocarcinoma
	221		poorly differentiated adenocarcinoma
**HPDE** ^{KECST2}	215	Spleen	poorly differentiated adenocarcinoma
	217		poorly differentiated adenocarcinoma
	218		moderate-to-poorly differentiated adenocarcinoma
**HPDE** ^{KCST2}	228		poorly differentiated adenocarcinoma
	229	Liver	poorly differentiated adenocarcinoma
	230		No tumors found

Supplementary Table 6. Gene Set Enrichment Analysis (GSEA) result

HALLMARK NAME	SIZE	NES	p-val	FDR q-val
EPITHELIAL_MESENCHYMAL_TRANSITION	161	1.78	0.0000	0.0164
G2M_CHECKPOINT	195	1.66	0.0000	0.0495
TNFA_SIGNALING_VIA_NFKB	187	1.63	0.0013	0.0467
IL6_JAK_STAT3_SIGNALING	66	1.61	0.0057	0.0437
COMPLEMENT	142	1.61	0.0027	0.0355
UV_RESPONSE_DN	132	1.59	0.0000	0.0336
SPERMATOGENESIS	76	1.58	0.0099	0.0341
MITOTIC_SPINDLE	198	1.53	0.0039	0.0483
APOPTOSIS	142	1.53	0.0095	0.0460

HALLMARK NAME = name of the individual hallmark geneset, SIZE = number of genes in each geneset, NES = Normalized Enrichment Score, p-val = nominal p-value, FDR = False Discovery Rate

Supplementary Table 7. PCR primer sequences

Purpose	ID	Fwd/Rev	sequence
lentiCRISPR cloning	Control	Forward	caccggtagcgaacgtgtccggcgt
lentiCRISPR cloning	Control	Reverse	aaacacgccggacacgttcgctacc
lentiCRISPR cloning	CDKN2A#1	Forward	caccgaccgtaactattcggtgcgt
lentiCRISPR cloning	CDKN2A#1	Reverse	aaacacgcaccgaatagttacggtc
lentiCRISPR cloning	SMAD4#1	Forward	caccgacaactcgttcgtagtgata
lentiCRISPR cloning	SMAD4#1	Reverse	aaactatcactacgaacgagttgtc
lentiCRISPR cloning	TP53#2	Forward	caccggggcagctacggtttccgtc
lentiCRISPR cloning	TP53#2	Reverse	aaacgacggaaaccgtagctgcccc
lentiCRISPR cloning	CDKN2A#3	Forward	caccggcatggagccttcggctgac
lentiCRISPR cloning	CDKN2A#3	Reverse	aaacgtcagccgaaggctccatgcc
lentiCRISPR cloning	SMAD4#2	Forward	caccgtacgaacgagttgtatcacc
lentiCRISPR cloning	SMAD4#2	Reverse	aaacggtgatacaactcgttcgtac
lentiCRISPR cloning	TP53#3	Forward	caccgaccagcagctcctacaccgg
lentiCRISPR cloning	TP53#3	Reverse	aaacccggtgtaggagctgctggtc
Transgene-specific PCR	KRAS	Forward	ctccgagcggatgtaccc
Transgene-specific PCR	KRAS	Reverse	cattgcactgtactcctctt
Transgene-specific PCR	CDKN2A#1	Forward	gagggcctatttcccatgatt
Transgene-specific PCR	CDKN2A#1	Reverse	aaacacgcaccgaatagttacggtc
Transgene-specific PCR	SMAD4#1	Forward	gagggcctatttcccatgatt
Transgene-specific PCR	SMAD4#1	Reverse	aaactatcactacgaacgagttgtc
Transgene-specific PCR	TP53#2	Forward	gagggcctatttcccatgatt
Transgene-specific PCR	TP53#2	Reverse	aaacgacggaaaccgtagctgcccc
Transgene-specific PCR	ERBB2	Forward	atgcggttttggcagtacat
Transgene-specific PCR	ERBB2	Reverse	cgagatctgagtccggtagc
Transgene-specific PCR	CDKN2A#3	Forward	gagggcctatttcccatgatt
Transgene-specific PCR	CDKN2A#3	Reverse	aaacgtcagccgaaggctccatgcc
Transgene-specific PCR	SMAD4#2	Forward	gagggcctatttcccatgatt
Transgene-specific PCR	SMAD4#2	Reverse	aaacggtgatacaactcgttcgtac
Transgene-specific PCR	TP53#3	Forward	gagggcctatttcccatgatt
Transgene-specific PCR	TP53#3	Reverse	aaacccggtgtaggagctgctggtc
TIDE PCR	CDKN2A#1 and CDKN2A#3	Forward	ggctcctcattcctcttcctt
TIDE PCR	CDKN2A#1 and CDKN2A#3	Reverse	cttgcctggaaagataccgc
TIDE PCR	SMAD4#1 and SMAD4#2	Forward	gcacaggccttgaaattatacc
TIDE PCR	SMAD4#1 and SMAD4#2	Reverse	ccttatttaaagtcgcgggcta
TIDE PCR	TP53#2 and TP53#3	Forward	gggtgtgatgggatggataaaag
TIDE PCR	TP53#2 and TP53#3	Reverse	ctgctcttttcacccatctacag
Off target analysis	CDKN2A#1, OFF1	Forward	gtgcacctgtaatccctgct
Off target analysis	CDKN2A#1, OFF1	Reverse	agggcttgggacataaagga
Off target analysis	CDKN2A#1, OFF2	Forward	attggtgaaccagggtgaaa
Off target analysis	CDKN2A#1, OFF2	Reverse	ttcaatggcctgtaaaattgg
Off target analysis	SMAD4#1, OFF1	Forward	ttttctcccccatctcagtg
Off target analysis	SMAD4#1, OFF1	Reverse	tggcttttaaggtgcattcc

Supplementary Note 1. Full unedited gel image for Figure 1E and 3B

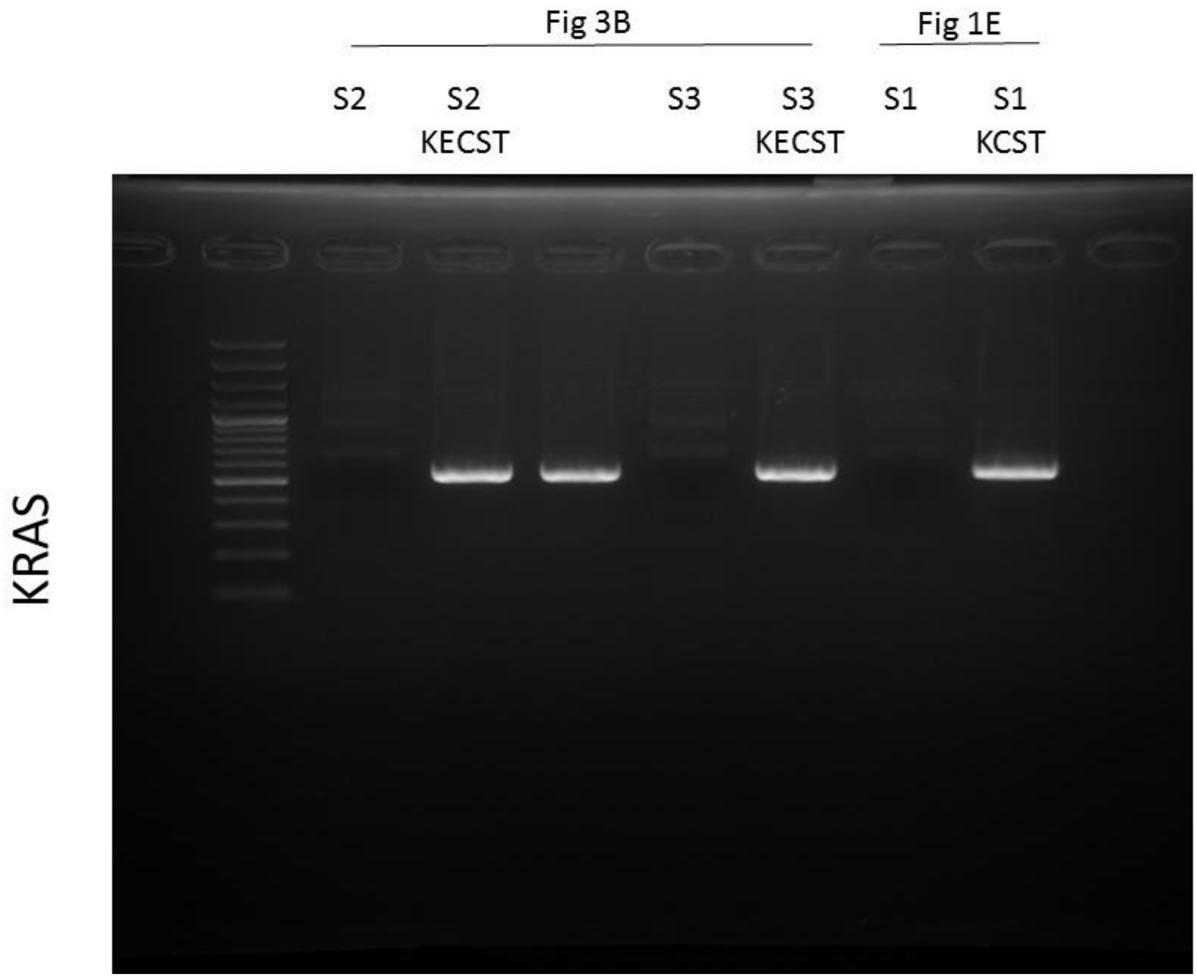

Supplementary Note 1. Full unedited gel image for Figure 1E and 3B (continued)

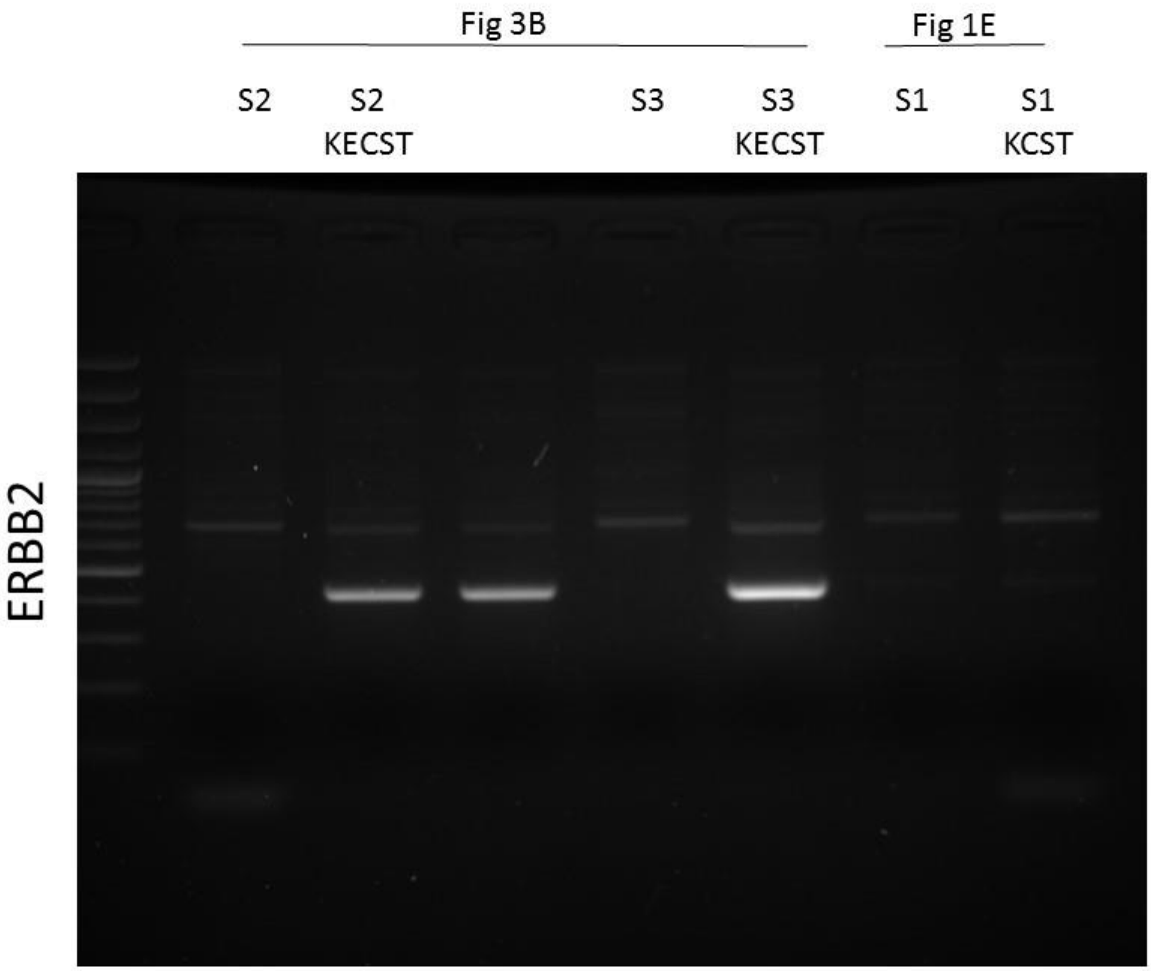

Supplementary Note 1. Full unedited gel image for Figure 1E and 3B (continued)

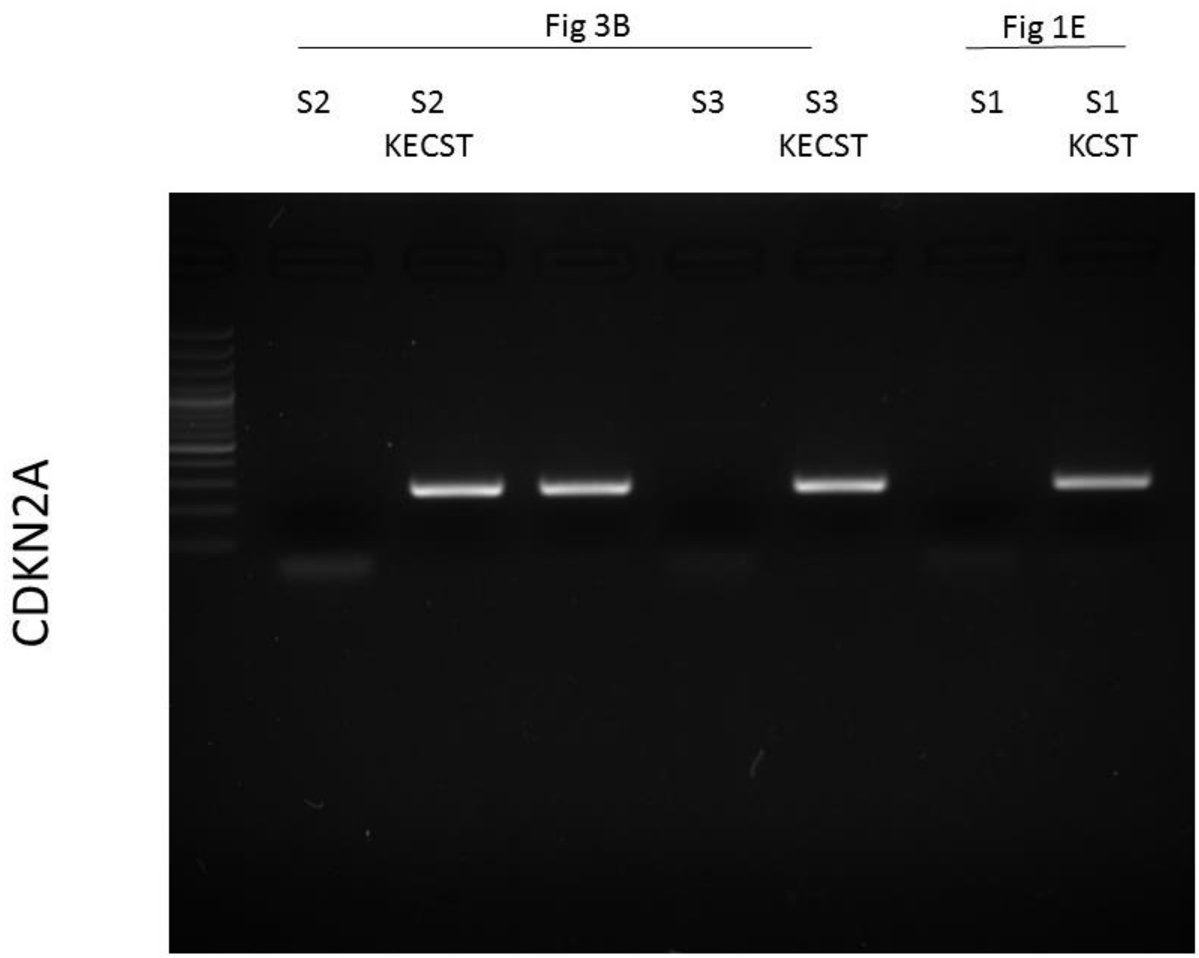

Supplementary Note 1. Full unedited gel image for Figure 1E and 3B (continued)

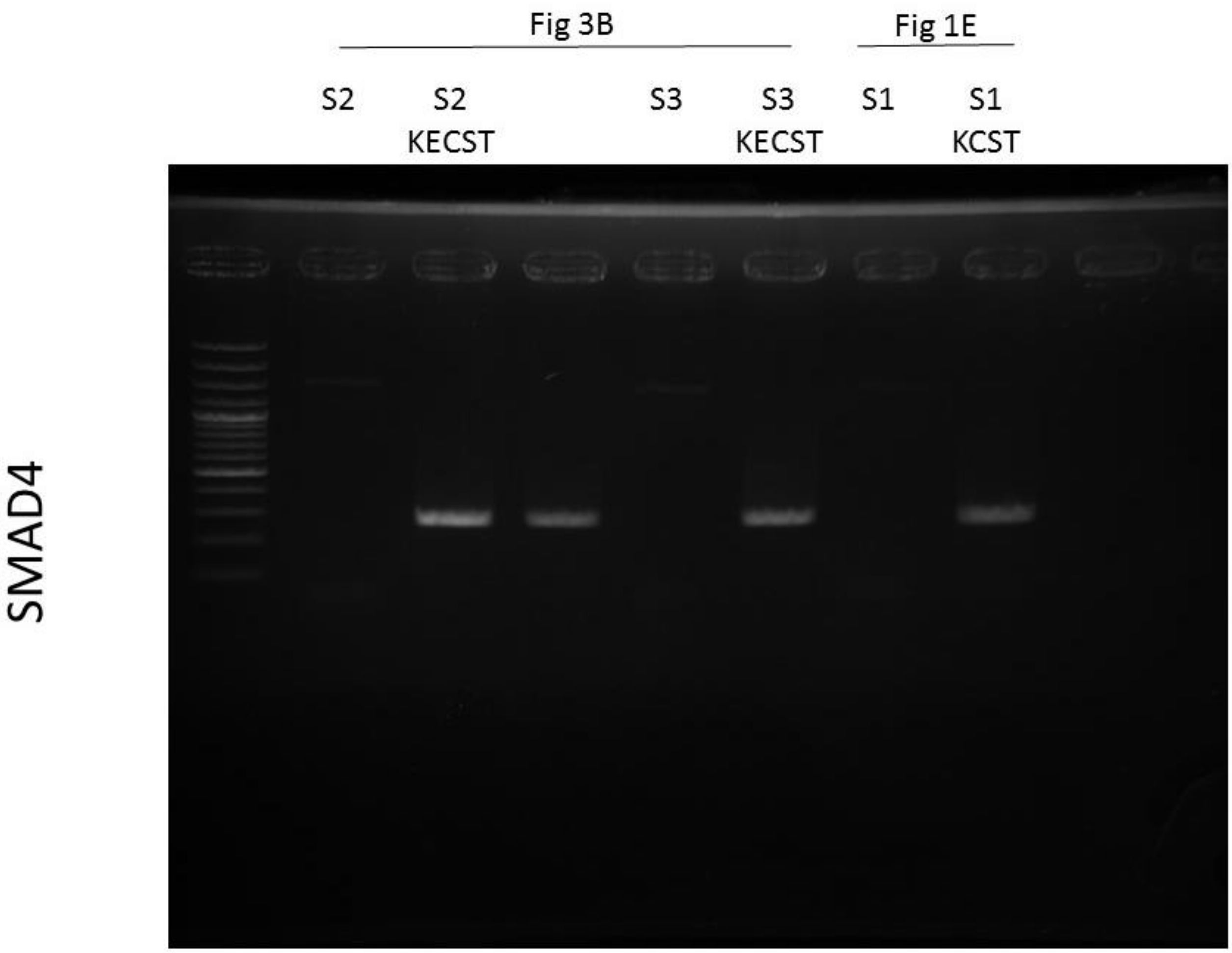

Supplementary Note 1. Full unedited gel image for Figure 1E and 3B (continued)

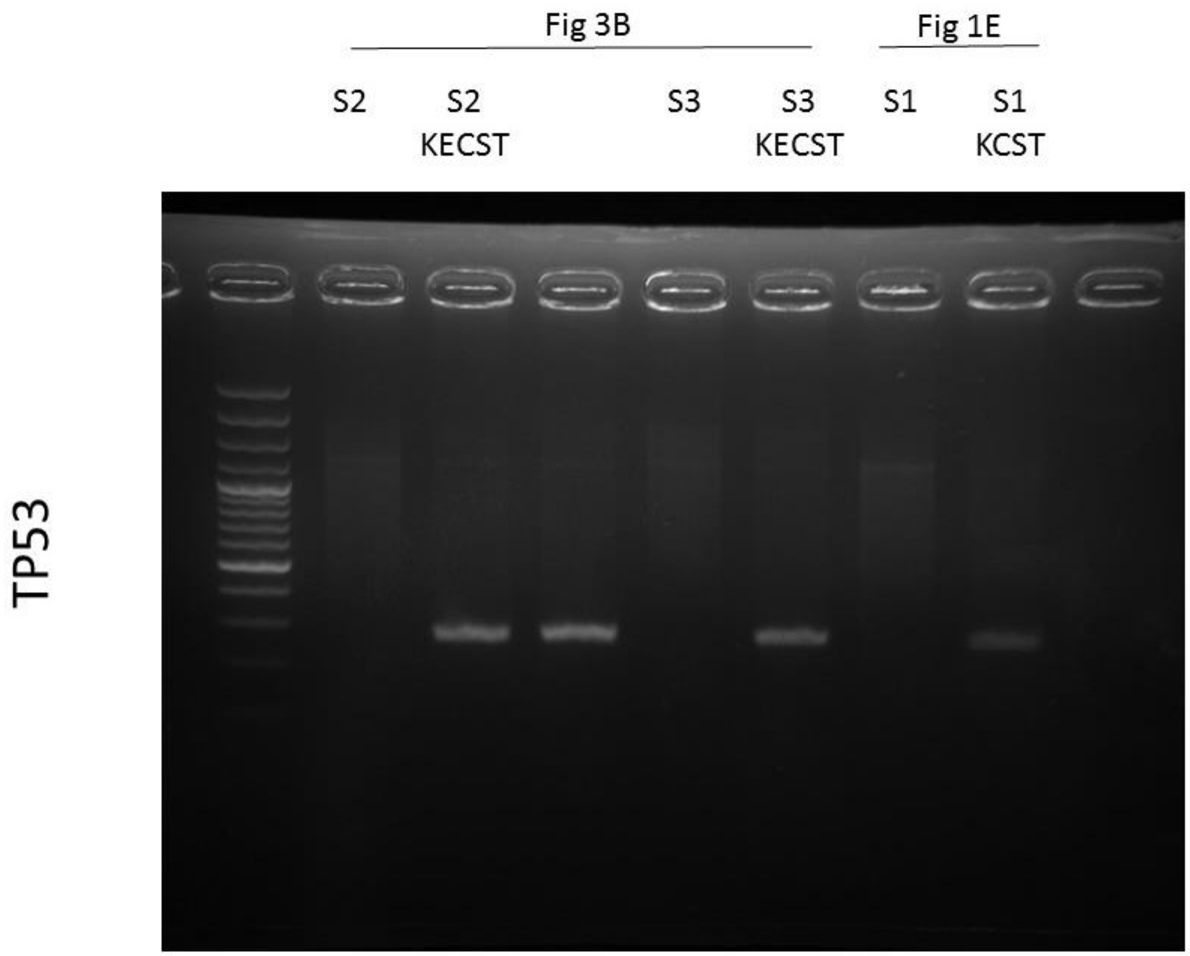

Supplementary Note 2. Full unedited gel image for Figure 5B and S1 sphere clones in Supplementary Figure 4E

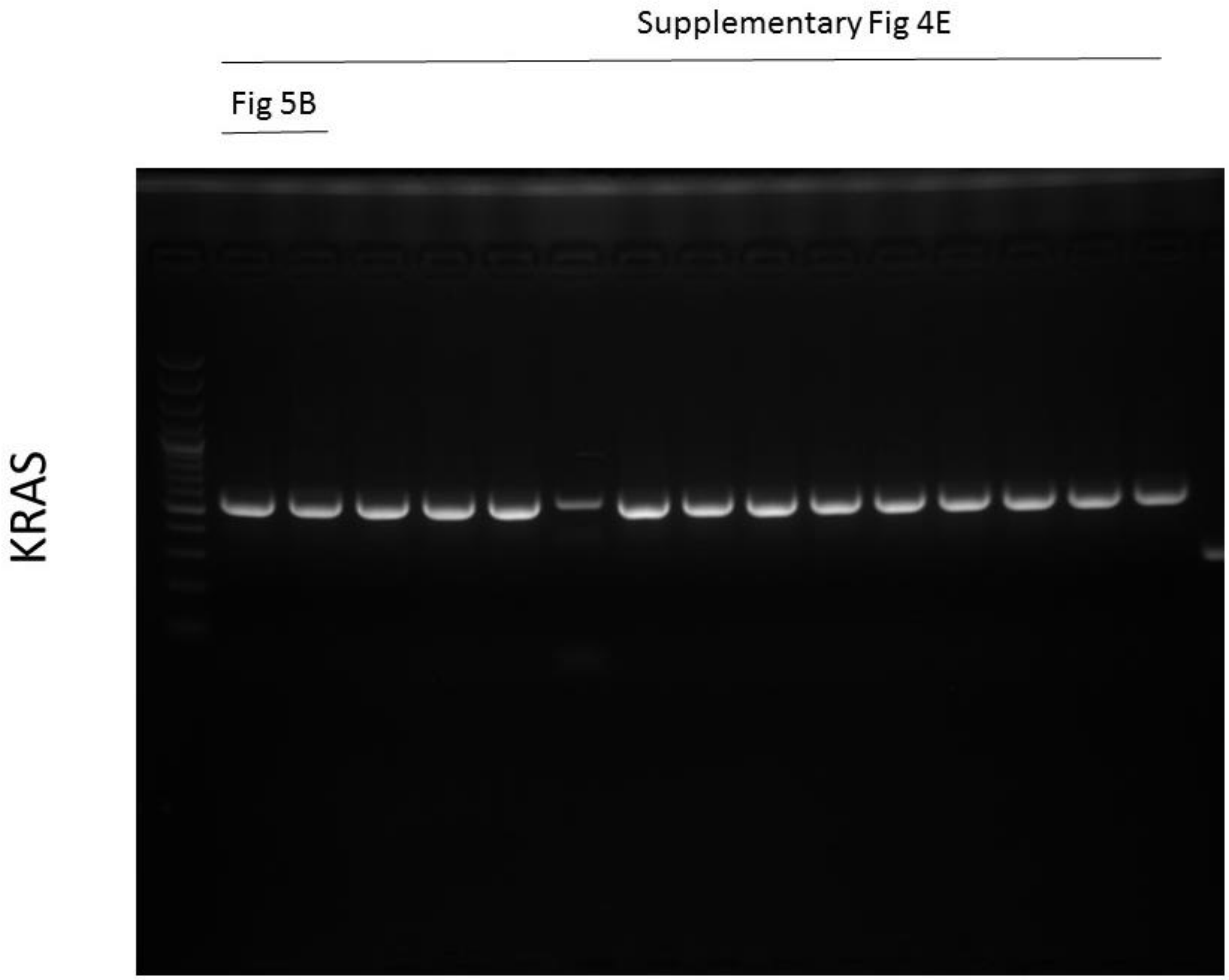

Supplementary Note 2. Full unedited gel image for Figure 5B and S1 sphere clones in Supplementary Figure 4E (continued)

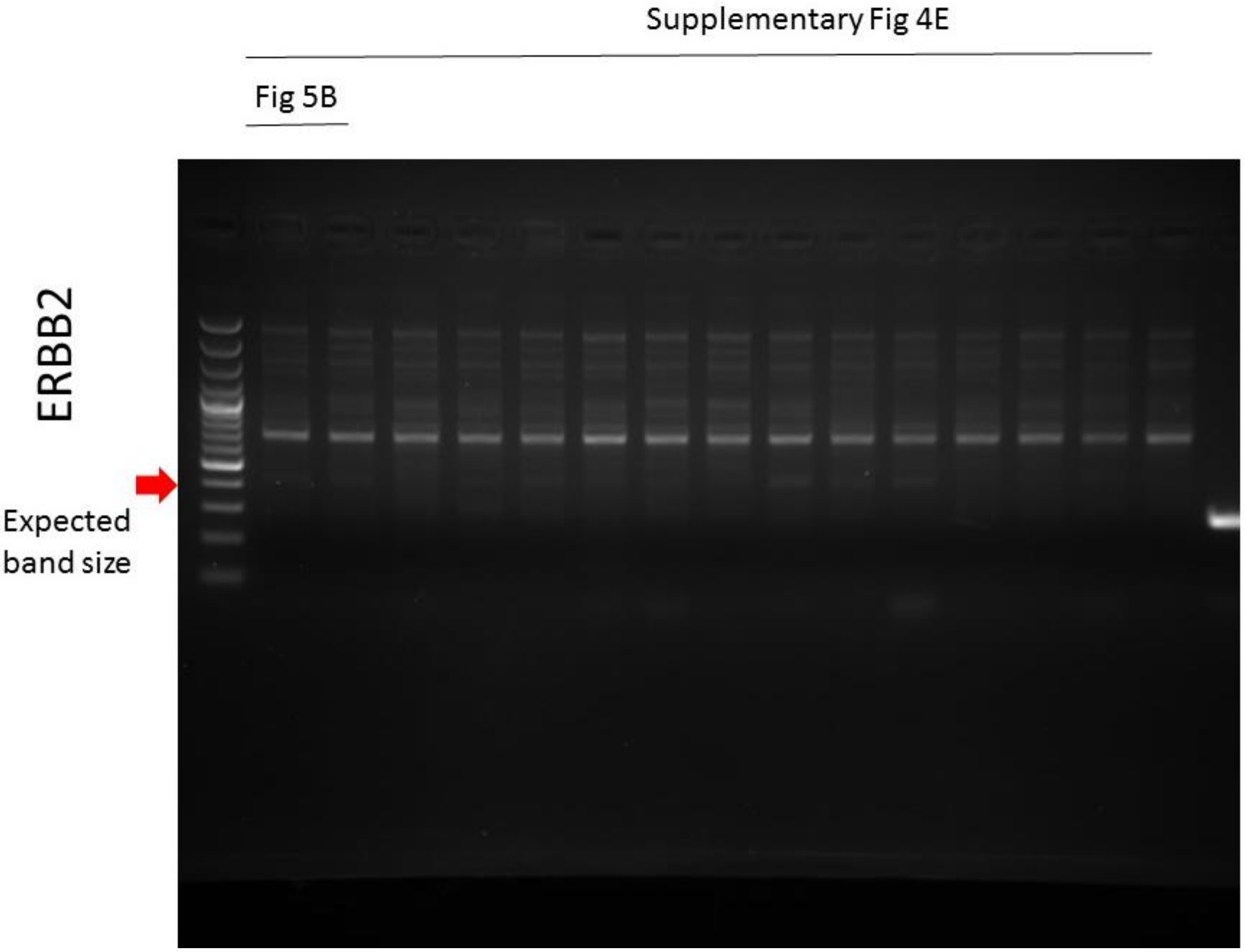

Supplementary Note 2. Full unedited gel image for Figure 5B and S1 sphere clones in Supplementary Figure 4E (continued)

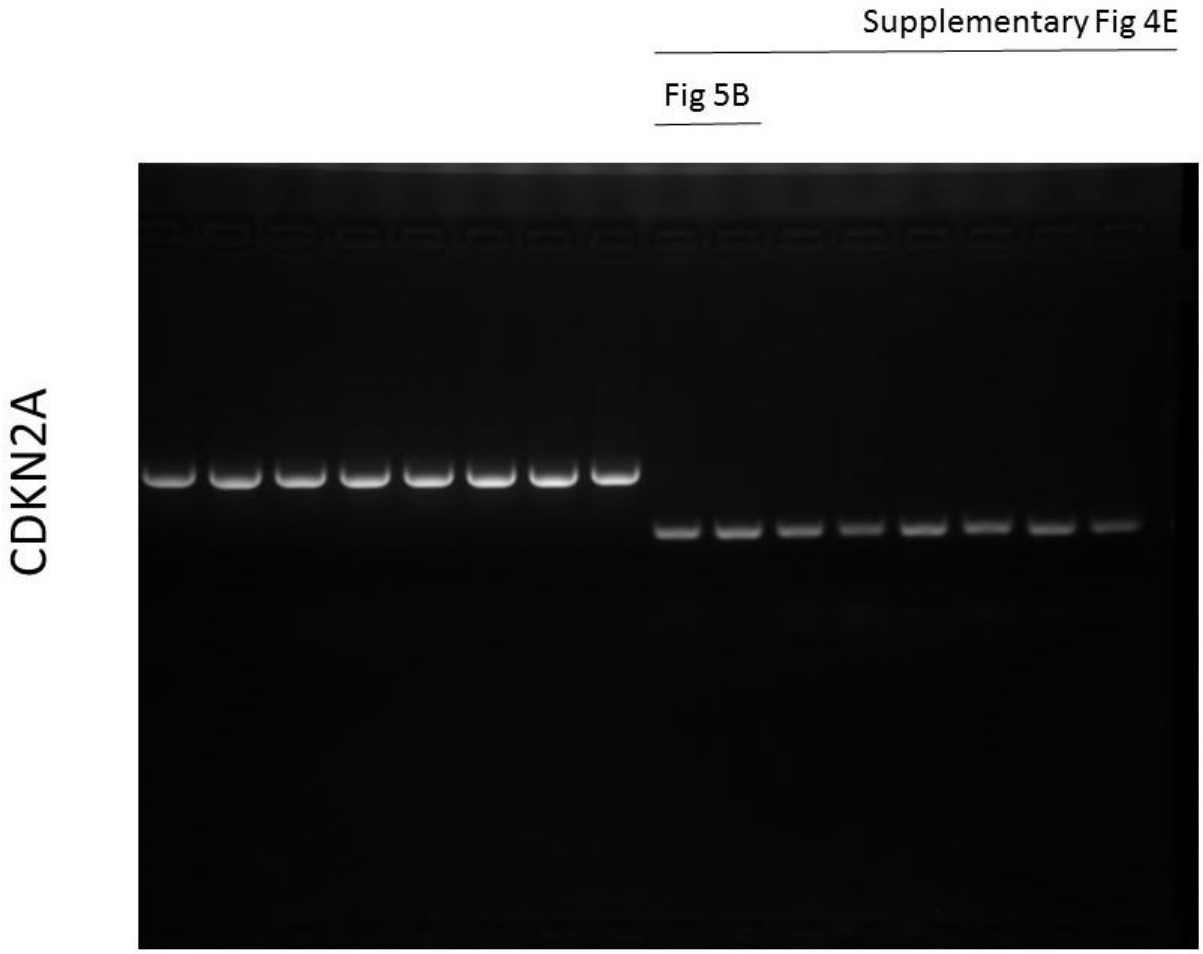

Supplementary Note 2. Full unedited gel image for Figure 5B and S1 sphere clones in Supplementary Figure 4E (continued)

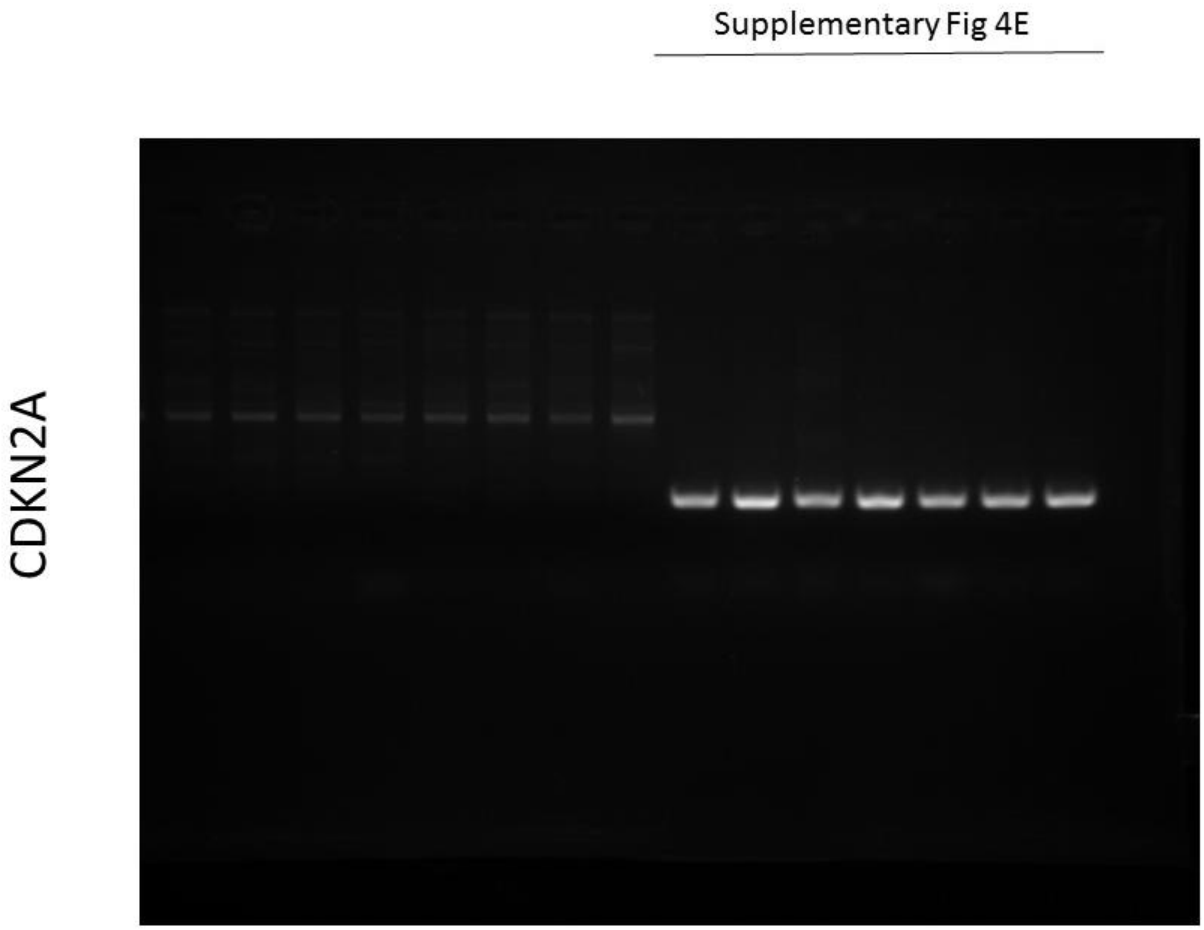

Supplementary Note 2. Full unedited gel image for Figure 5B and S1 sphere clones in Supplementary Figure 4E (continued)

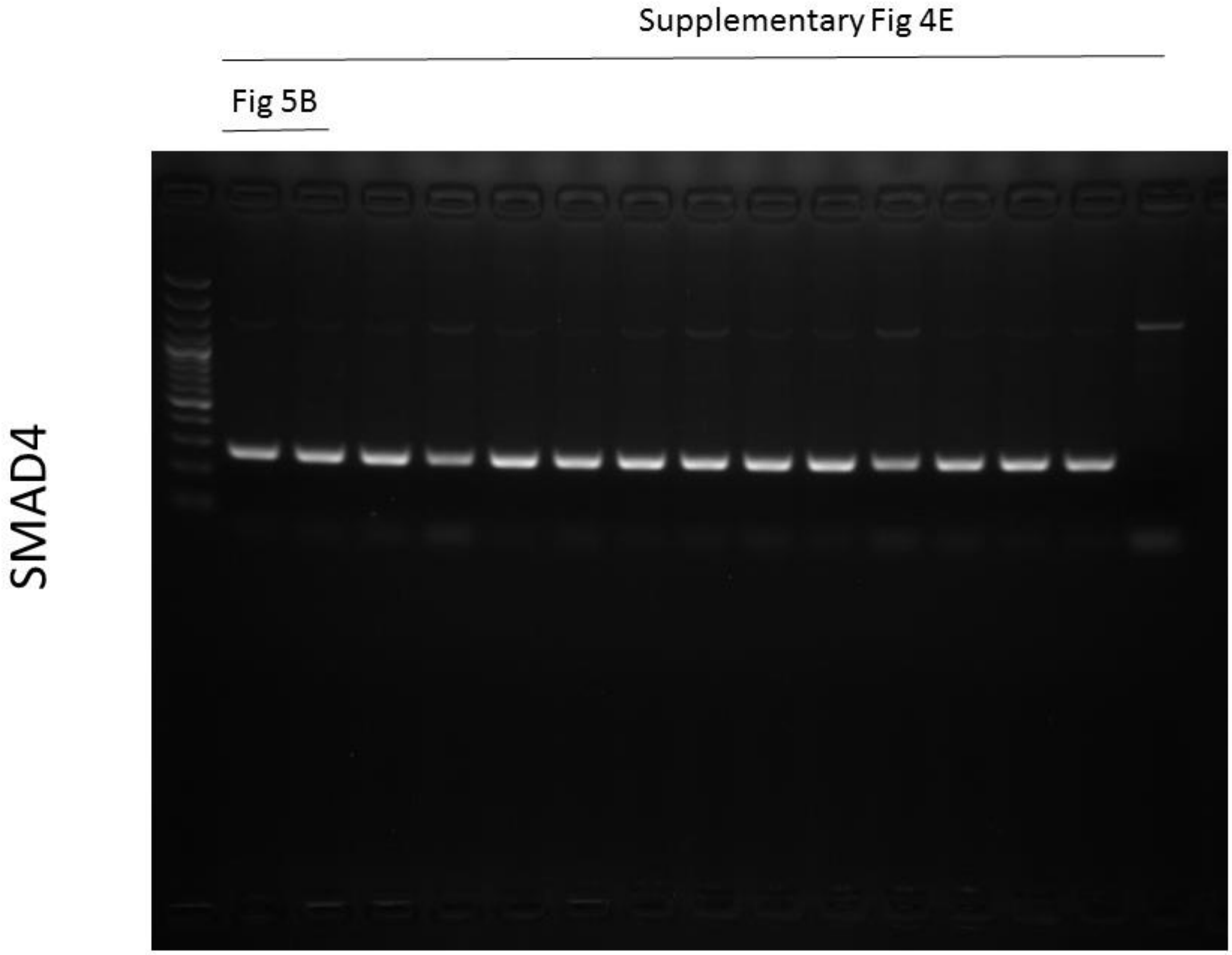

Supplementary Note 2. Full unedited gel image for Figure 5B and S1 sphere clones in Supplementary Figure 4E (continued)

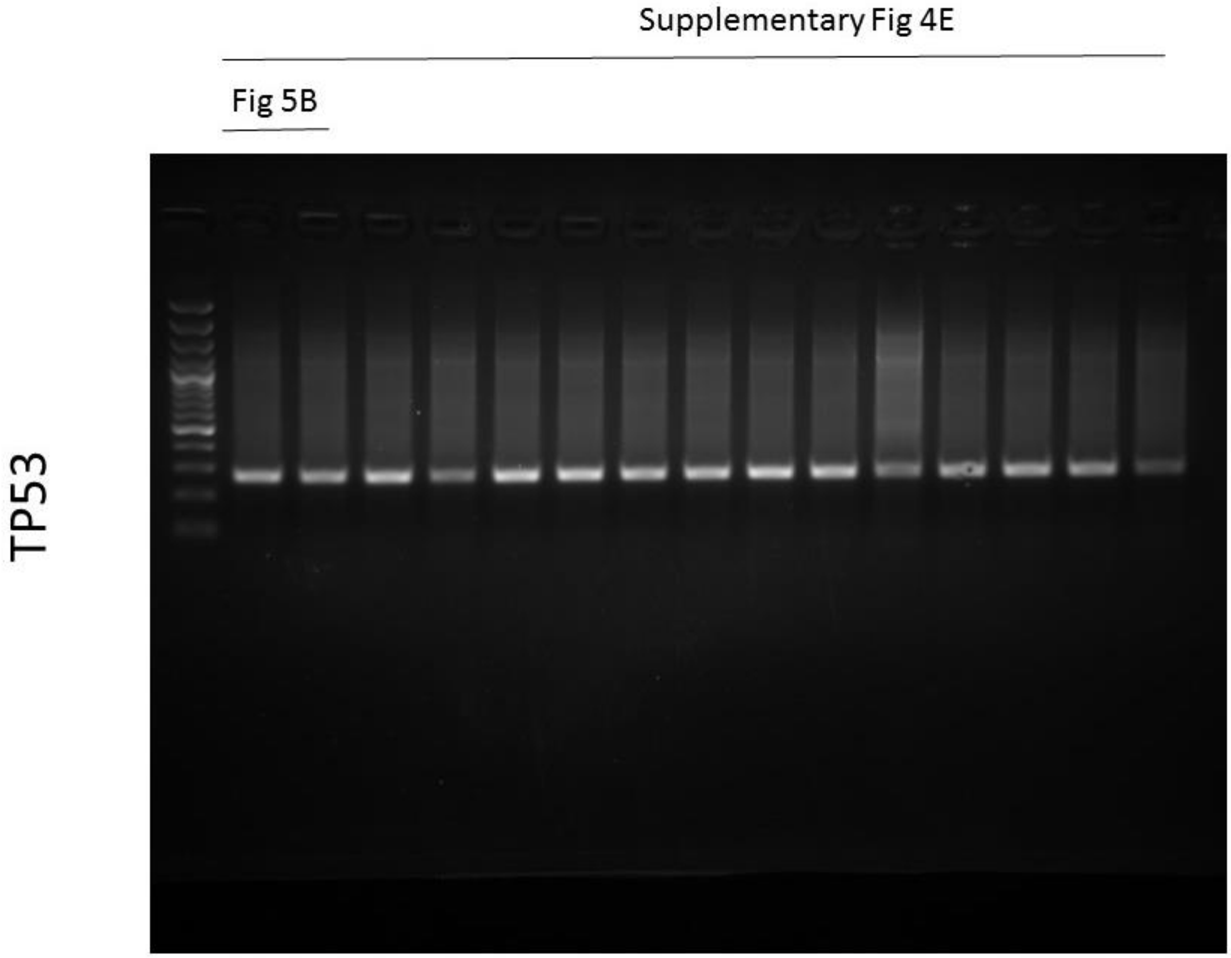

Supplementary Note 3. Full unedited gel image for S2 and S3 sphere clones in Supplementary Figure 4E

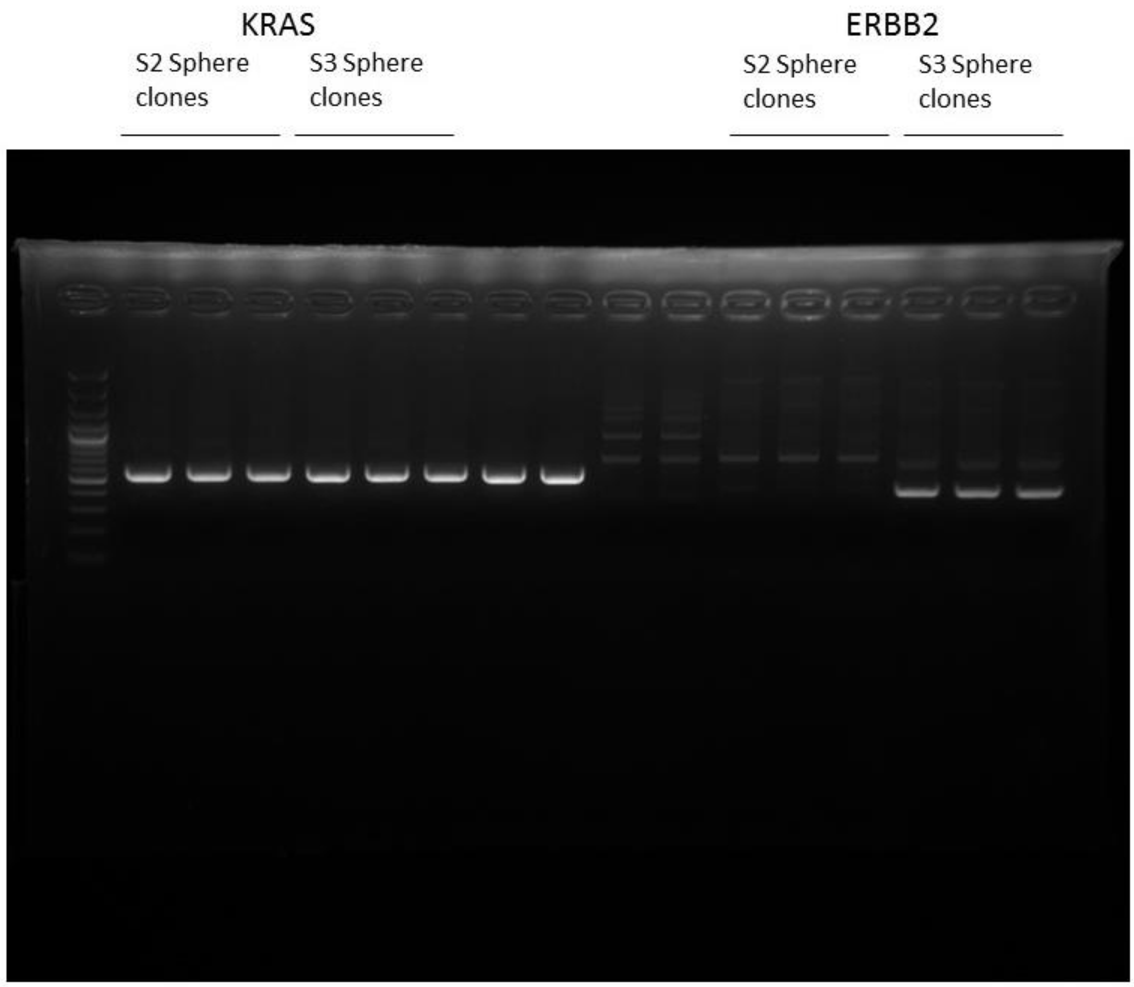

Supplementary Note 3. Full unedited gel image for S2 and S3 sphere clones in Supplementary Figure 4E (continued)

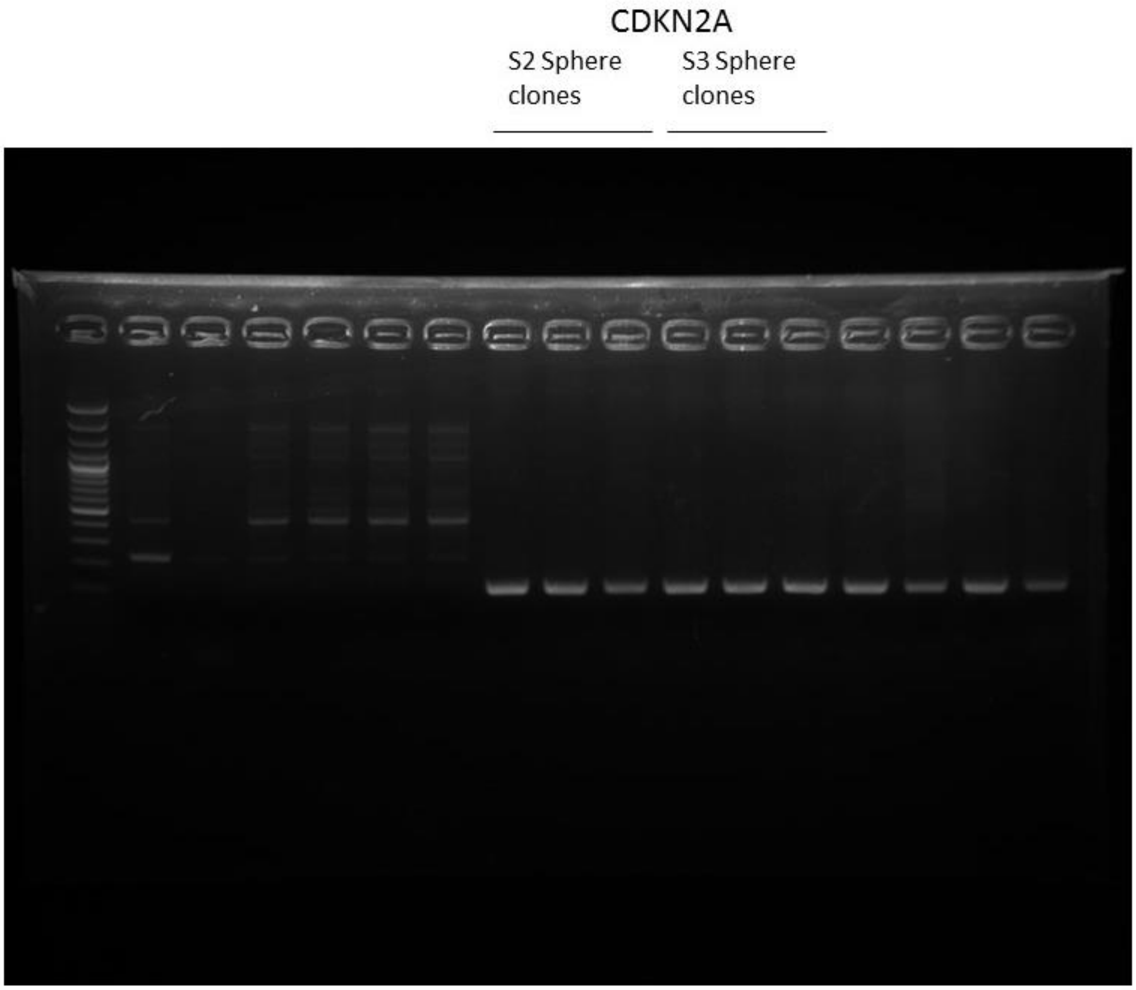

Supplementary Note 3. Full unedited gel image for S2 and S3 sphere clones in Supplementary Figure 4E (continued)

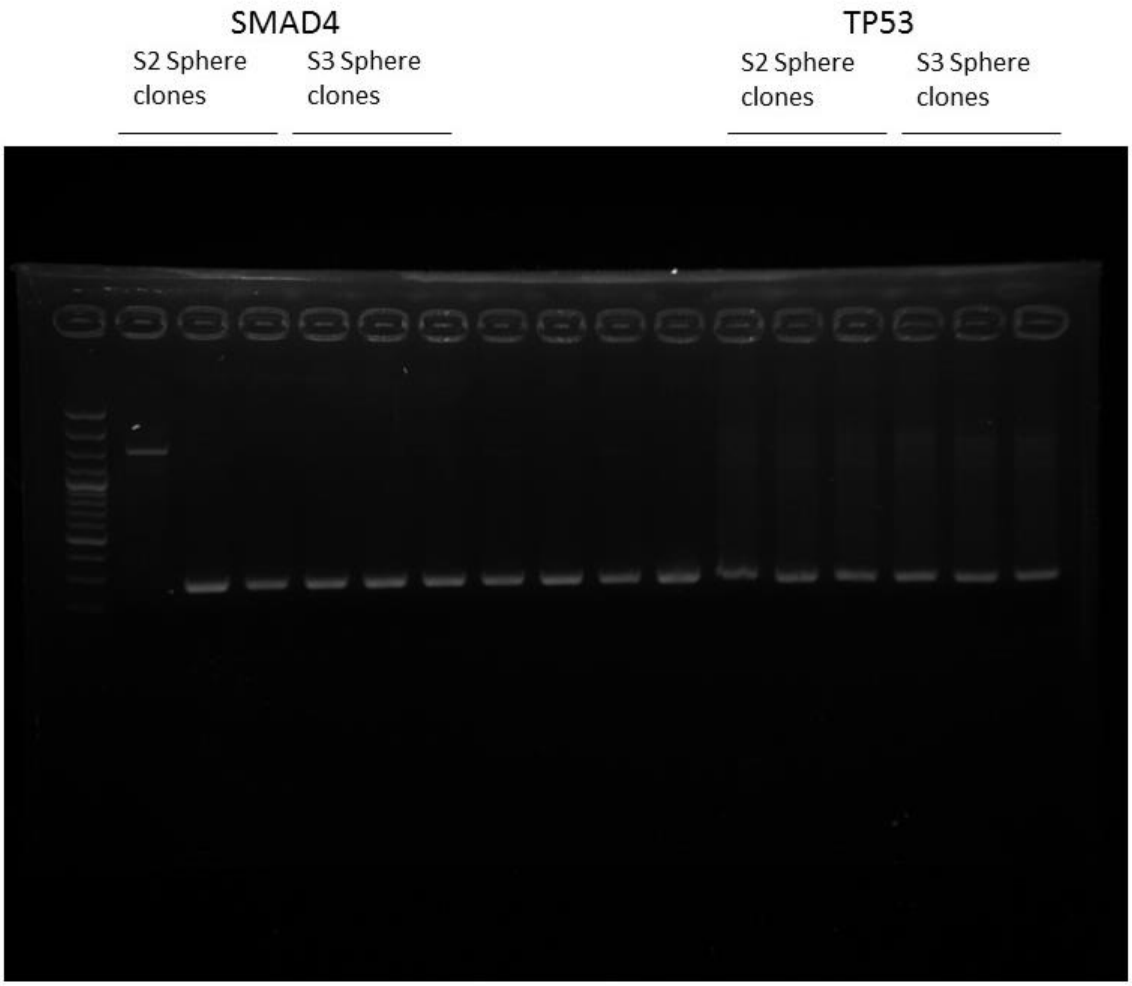

Supplementary Note 4. Full unedited gel image for Figure 6A

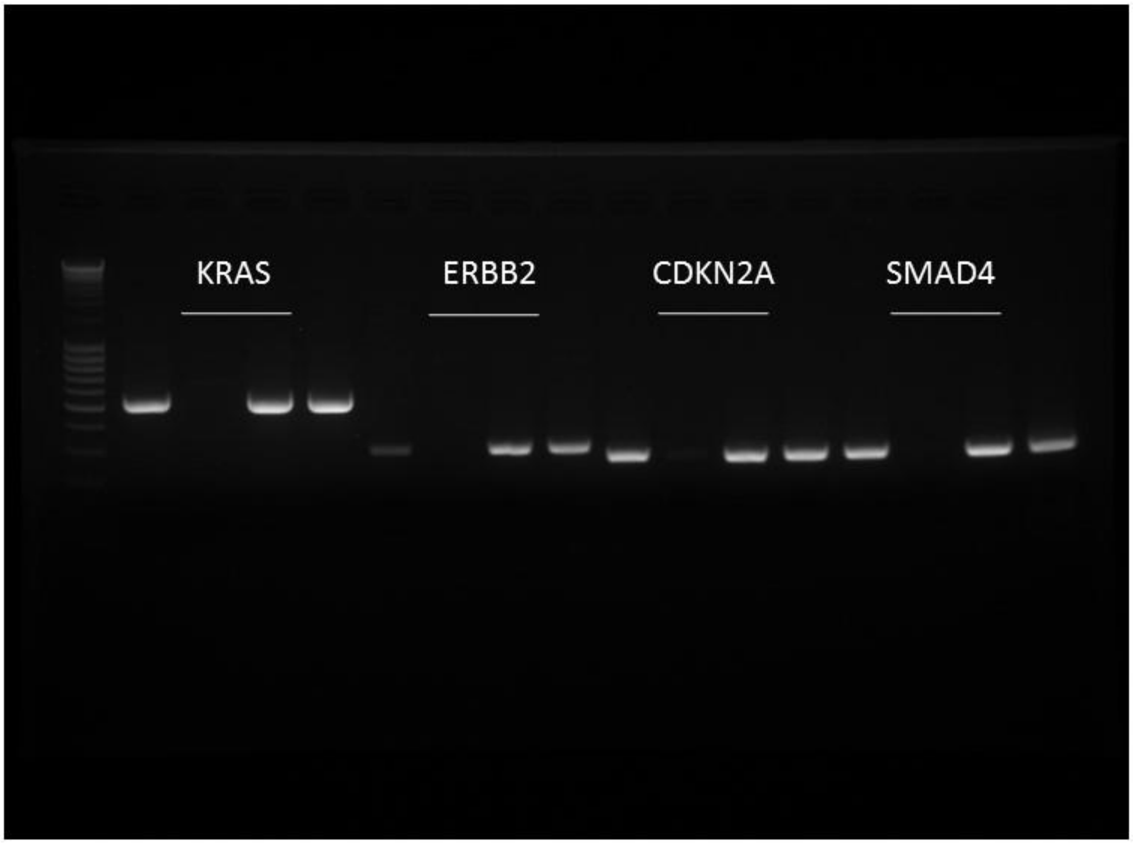

Supplementary Note 4. Full unedited gel image for Figure 6A (continued)

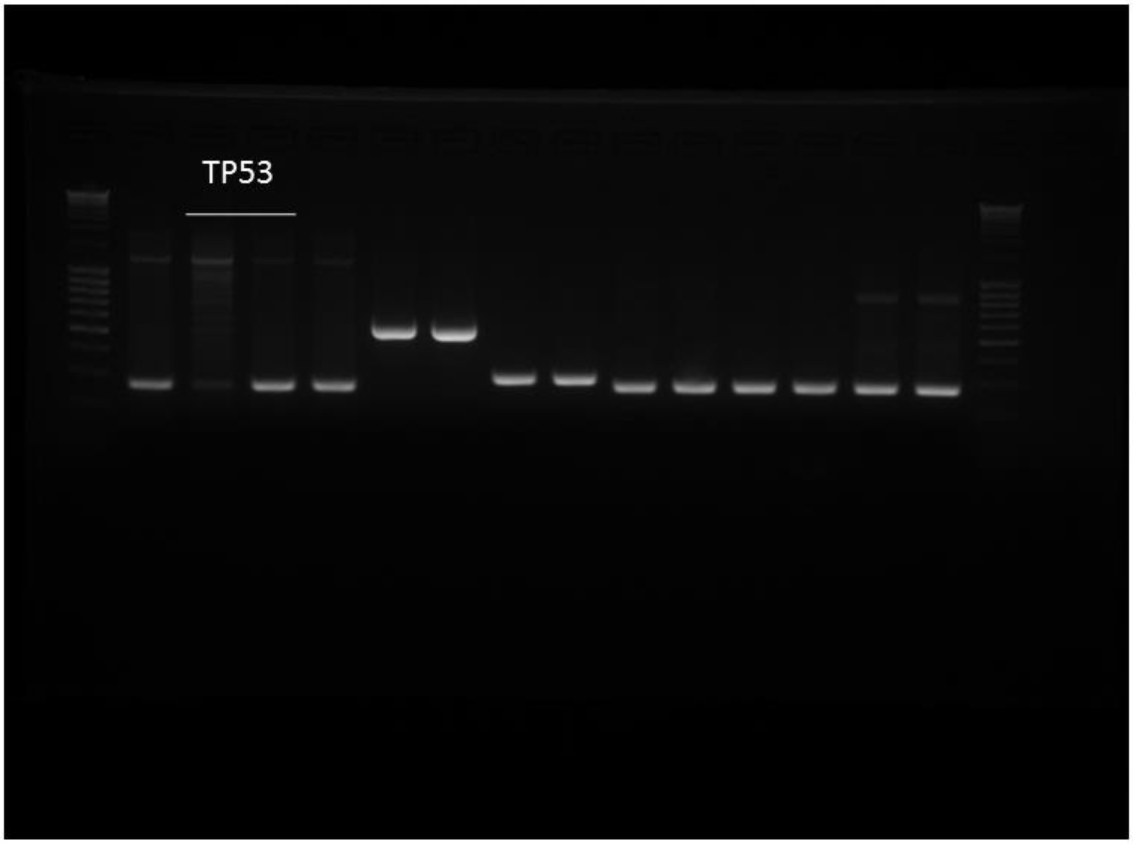

Supplementary Note 5. Full unedited gel image for Supplementary Figure 15C

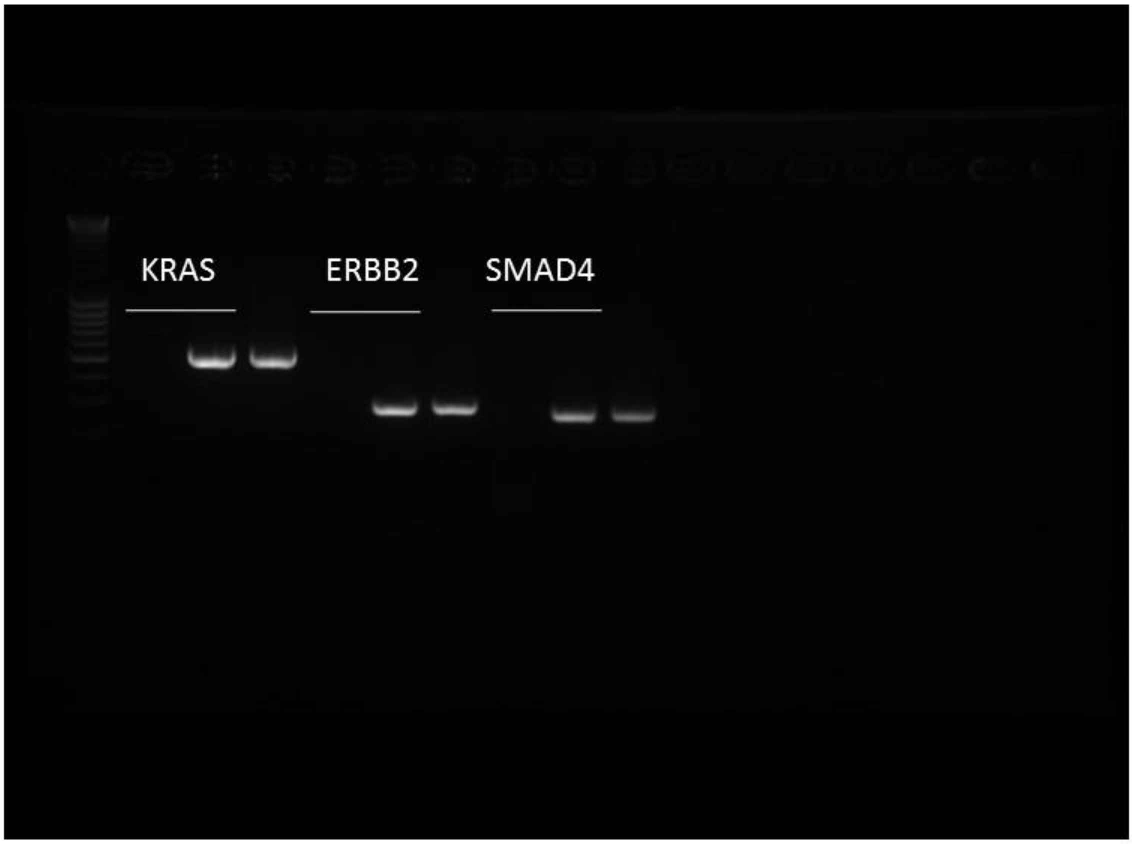

Supplementary Note 5. Full unedited gel image for Supplementary Figure 15C (continued)

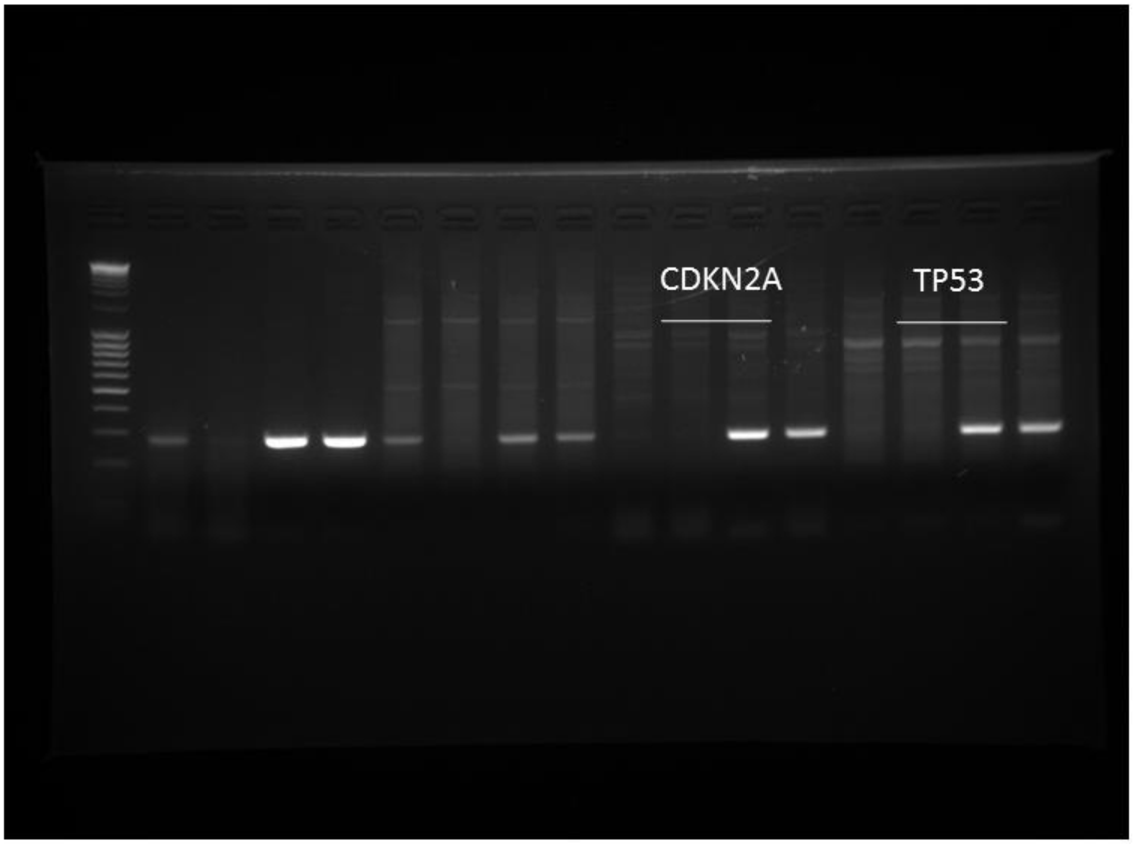

Supplement: Supplementary Information — Supplementary Figures, Supplementary Tables, Supplementary Notes. [file ncomms14686-s1.pdf]
